# Comparison of 6-month outcomes of sepsis versus non-sepsis critically ill patients receiving mechanical ventilation

**DOI:** 10.1186/s13054-022-04041-w

**Published:** 2022-06-13

**Authors:** Carol L. Hodgson, Alisa M. Higgins, Michael Bailey, Jonathon Barrett, Rinaldo Bellomo, D. James Cooper, Belinda J. Gabbe, Theodore Iwashyna, Natalie Linke, Paul S. Myles, Michelle Paton, Steve Philpot, Mark Shulman, Meredith Young, Ary Serpa Neto, The PREDICT Study Investigators

**Affiliations:** 1 Australian and New Zealand Intensive Care Research Centre, School of Public Health and Preventive Medicine, Monash University, 553 St Kilda Rd, Melbourne, VIC 3004 Australia; 2 Department of Intensive Care and Hyperbaric Medicine, The Alfred, Melbourne, VIC Australia; 3 Intensive Care Unit, Epworth Healthcare, Melbourne, VIC Australia; 4 Faculty of Medicine, Nursing and Health Sciences, Monash University, Melbourne, VIC Australia; 5 Department of Critical Care, University of Melbourne, Melbourne, VIC Australia; 6 Department of Intensive Care, Austin Health, Melbourne, VIC Australia; 7 School of Public Health and Preventive Medicine, Monash University, Melbourne, VIC Australia; 8 Division of Pulmonary and Critical Care, Department of Medicine, University of Michigan, Ann Arbor, USA; 9 Centre for Clinical Management Research, VA Ann Arbor Healthcare System, Ann Arbor, MI USA; 10 Department of Anaesthesiology and Perioperative Medicine, The Alfred, Melbourne, VIC Australia; 11 Department of Physiotherapy, Monash Health, Melbourne, VIC Australia; 12 Intensive Care Unit, Cabrini Health, Melbourne, VIC Australia; 13 Department of Critical Care Medicine, Hospital Israelita Albert Einstein, São Paulo, Brazil

**Keywords:** Intensive care, Sepsis, Disability, Recovery, Mechanical ventilation, Critical illness

## Abstract

**Background:**

Data on long-term outcomes after sepsis-associated critical illness have mostly come from small cohort studies, with no information about the incidence of new disability. We investigated whether sepsis-associated critical illness was independently associated with new disability at 6 months after ICU admission compared with other types of critical illness.

**Methods:**

We conducted a secondary analysis of a multicenter, prospective cohort study in six metropolitan intensive care units in Australia. Adult patients were eligible if they had been admitted to the ICU and received more than 24 h of mechanical ventilation. There was no intervention.

**Results:**

The primary outcome was new disability measured with the WHO Disability Assessment Schedule 2.0 (WHODAS) 12 level score compared between baseline and 6 months. Between enrollment and follow-up at 6 months, 222/888 (25%) patients died, 100 (35.5%) with sepsis and 122 (20.1%) without sepsis (*P* < 0.001). Among survivors, there was no difference for the incidence of new disability at 6 months with or without sepsis, 42/106 (39.6%) and 106/300 (35.3%) (RD, 0.00 (− 10.29 to 10.40), *P* = 0.995), respectively. In addition, there was no difference in the severity of disability, health-related quality of life, anxiety and depression, post-traumatic stress, return to work, financial distress or cognitive function.

**Conclusions:**

Compared to mechanically ventilated patients of similar acuity and length of stay without sepsis, patients with sepsis admitted to ICU have an increased risk of death, but survivors have a similar risk of new disability at 6 months.

*Trial registration* NCT03226912, registered July 24, 2017.

**Supplementary Information:**

The online version contains supplementary material available at 10.1186/s13054-022-04041-w.

## Introduction

Sepsis is a dysregulated host response to infection that leads to multi-organ failure and, in many patients, death or disability [1, 2]. It caused one in five of all global deaths in 2017 (11 million deaths/48.9 million cases) and is one of the most common complications amongst COVID-19 patients [3, 4]. Among survivors of sepsis, poor long-term outcomes have been reported, including increased hospital readmissions, functional impairment and reduced health-related quality of life [5].

As recommendations accrue for ICU interventions to improve long-term outcomes, it is important to identify appropriate target groups. Currently, it is unclear in critically ill patients whether the response to infection in patients with sepsis leads to worse 6-month functional outcomes compared to patients with a similar severity of illness without sepsis. We recently reported a registry-embedded, multicenter, prospective cohort study to assess long-term outcomes in a diverse population of critically ill patients [6, 7]. In the present study, we compared the outcomes at 3 and 6 months of mechanically ventilated patients with and without sepsis. We hypothesized that the presence of sepsis during the ICU stay would be associated with greater burden of new disability in survivors at 6 months.

## Methods

### Study design

This was a secondary analysis of a registry-embedded, prospective, multicenter, longitudinal cohort study conducted in six metropolitan ICUs in the State of Victoria, Australia. It included four public tertiary teaching hospitals and two private hospitals (Additional file 1: Table S1). More information on study design is available [6, 7]. Ethics committee approval, including a waiver of consent for hospital data and an opt-out consent for follow-up data, was obtained at each site under a national mutual acceptance scheme for sites (NMA Reference No. HREC/17/MonH/217). Verbal consent was confirmed by telephone at the start of the first interview.

### Participants

Consecutive eligible patients were identified from the hospital clinical information system at each participating site. Patients were eligible if they had been admitted to the ICU and had received more than 24 h of mechanical ventilation. Patients were excluded if they were aged less than 18 years old, had a proven or suspected acute primary brain process that was likely to result in global impairment of consciousness or cognition (e.g., traumatic brain injury or stroke) or did not speak English.

### Data collection

Demographic, intervention and hospital outcome data were obtained from electronic health records for all eligible patients under a waiver of consent. Data from any second or subsequent readmission to ICU during the index hospital admission were excluded. Patients who survived the hospital admission were contacted by mail and invited to participate in telephone interviews at 3 and 6 months after ICU admission.

Patient-reported long-term outcomes were assessed at two pre-specified time points. Baseline health and disability (defined as the status one month before ICU admission) were assessed retrospectively at the 3-month interview. All assessments were performed by telephone by trained central outcome assessors located at Monash University, who were blinded to the details of the patient’s hospital admission. Data were entered into an electronic data capture system (REDCap®, Vanderbilt University, Tennessee, USA). Patients were followed from ICU admission until 6 months or death, whichever occurred first.

### Definitions

The exposure of interest was the presence of sepsis during ICU stay. This applied either to patients admitted with the diagnosis of sepsis or who developed sepsis at any time during the ICU admission. Sepsis at admission was identified according to APACHE III codes, and sepsis during the ICU stay was identified using hospital coding for sepsis (ICD 10 AM coding A41: 501 sepsis other than urinary, 502 sepsis of urinary tract origin, 503 sepsis with shock other than urinary, 504 sepsis of urinary tract origin with shock), as is mandatorily reported by the hospitals.

The overall population of the study was defined as the ‘Hospital Cohort.’ The main population of interest for the primary analysis of this manuscript comprises patients with available outcomes at 6 months (defined as the ‘Follow-Up Cohort’).

### Outcomes

The primary outcome was new disability measured with the WHO Disability Assessment Schedule 2.0 (WHODAS) 12 level score compared between baseline and 6 months. WHODAS 12 level score, assessed in its original scale in a continuous form (ranging from 0 to 100%) [8]. The WHODAS has been validated in the ICU population [9]. Secondary outcomes included the assessment of the following domains: (a) health status at 3 and 6 months (measured by EQ-5D-5L visual analogue scale [VAS] and utility score) [8], (b) anxiety and depression at 6 months (measured by the Hospital Anxiety and Depression Scale, HADS) [8, 10], (c) return to work at 3 and 6 months (measured by WHODAS), (d) post-traumatic stress disorder at 6 months (measured by the Impact of Event Scale–Revised, IES-R [ranging from 0 to 88]) [8, 11]; (e) daily activities at 6 months (measured by the Instrumental Activities of Daily Living, IADL [ranging from 0 to 8]) [12]; cognitive function at 6 months (measured by the Montreal Cognitive Assessment, MoCA-BLIND [ranging from 0 to 22]) [6, 13, 14]; and (f) financial distress at 3 and 6 months (assessed through a scale from 0 to 10, with 0 as the minimum stress and 10 the maximum). In addition to the assessment of the outcomes on a continuous scale, pre-defined categories were also assessed at 3 and 6 months [7, 8].

Severity of illness was collected at ICU admission, and physiological variables were the worst value within the first 24 h of ICU admission. Clinical outcomes such as duration of ventilation, ICU and hospital length of stay and ICU, hospital, 90- and 180-day mortality were also reported.

### Management of missing data

Because missing data rarely occur entirely at random, the association between characteristics of the patients and status with respect to missing data was assessed [15, 16]. Missing data in baseline characteristics and long-term outcomes are reported in Additional file 1: Tables S2 and S3.

The main analysis was conducted using all available data and in the population of patients without missing data for the proposed outcome. A sensitivity analysis for missingness and truncation due to death was pre-specified and performed for each outcome. The sensitivity analysis considered a multiple imputation of missing long-term outcomes in patients known to be alive at the time point of measurement (described in Additional file 1: Methods). We used all patients available with outcomes at 3 and 6 months.

Based on standard deviation in WHODAS of 21%(10), with a minimum of 200 patients per group, this study will have > 99% power (2-sided *p* value of 0.05) to detect a minimum clinically significant difference in WHODAS of 10% between groups. These calculations include 15% inflation for potential non-normality in WHODAS.

### Statistical analysis

Continuous variables are reported as medians and interquartile ranges (IQR) and categorical variables as number and percentage. Comparison between groups were done using a Wilcoxon rank-sum test for continuous variables and Fisher exact test for categorical variables. 6-month survival was reported in a Kaplan–Meier curve and compared between the groups using a log-rank test.

Long-term outcomes reported at more than one time point were assessed with a mixed-effects generalized linear model considering a Gaussian distribution including all post-baseline assessments, and with time of measurement (at 3 and 6 months), group (sepsis or no sepsis), as well as the group x time interaction as a fixed effect. The time of measurement was treated as a categorical variable and random intercepts for patients and centers were included to account for the dependency of repeated measures and clustering of the data. Between-group comparisons at each time point were estimated with the appropriate contrasts from the model and using a Holm–Bonferroni method to adjust for multiplicity. Binary outcomes were assessed using the same strategy but considering a binomial distribution with an identity link. Long-term outcomes reported only at 6 months were compared using a simple mixed-effects generalized linear model considering a Gaussian or binomial distribution (with an identity link) with group as fixed effect and centers included as random effect. All results are presented as absolute differences with 95% confidence intervals.

To adjust for baseline imbalances, the following covariates were included as fixed effect in the models described above: age, sex, ICU admission source, APACHE III score, type of admission (medical vs. surgical), lung transplant patients, trauma, creatinine, heart rate, mean arterial pressure, presence of chronic cardiovascular disease and ICU length of stay. These variables were selected a priori and based on clinical relevance only as described in previous reports [17]. Whenever available, models were further adjusted by the baseline value of the outcome of interest as a fixed effect. Sensitivity analyses due to missing data are described above.

All analyses were performed considering a two-sided hypothesis test, with a significance level of 0.01 to compensate for multiplicity. Analyses were performed using the software R v.4.0.3 (R Core Team, 2016, Vienna, Austria) [18].

## Results

### Population

We screened 1475 patients, admitted to ICU between May 2017 and June 2018. Of the 899 patients who met eligibility criteria, 888 were enrolled, 282 with sepsis (Additional file 1: Figure S1). Patients without sepsis are described in Additional file 1: Table S4. Between enrollment and the 6-month follow-up, 222 (25%) patients died (100 [35.5%] in the sepsis group and 122 [20.1%] in the non-sepsis group), and 66 (7%) opted-out of the 3 and/or 6-month telephone follow-up. Of the 888 patients enrolled in the study, 670 (75.5%) had outcomes of death or disability available at 6 months. We conducted the final 6-month follow-up on January 23, 2019. Missing data are shown in Additional file 1: Tables S2 and S3.

Characteristics of patients in the ‘Follow-Up Cohort’ and of patients who died before 6 months are shown in Additional file 1: Table S5. Characteristics of the patients who responded to the follow-up interviews at 6 months and of the patients who did not respond are shown in Additional file 1: Table S6.

**Fig. 1:**
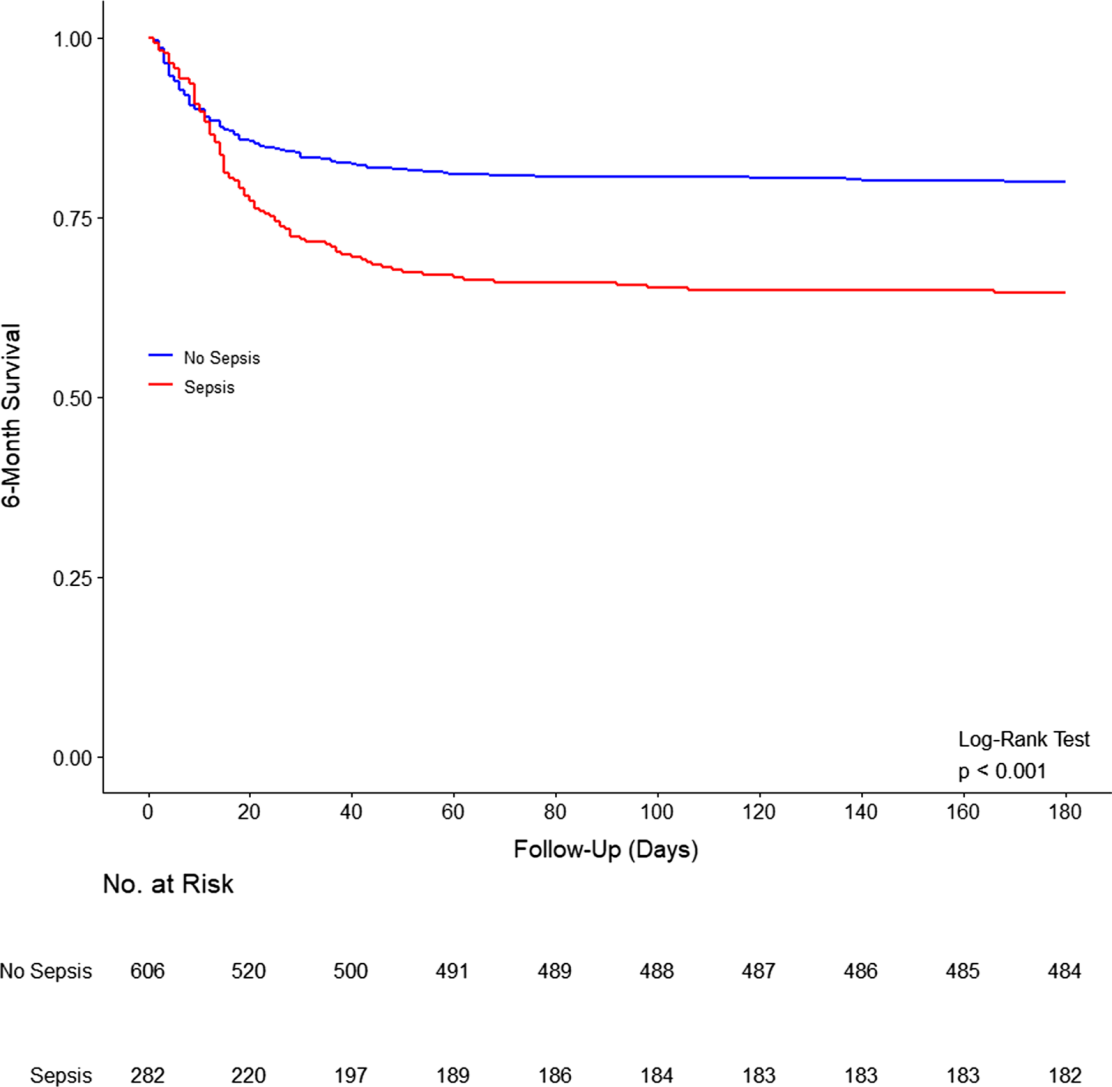
Kaplan–Meier curve of 6-month survival in patients with sepsis (red) and without sepsis (blue)

At ICU admission, patients with sepsis had a higher APACHE III scores, a higher proportion had acute respiratory failure, and a lower proportion were separated or divorced (Table 1). Need for renal replacement therapy, extracorporeal membrane oxygenation, noninvasive ventilation, tracheostomy and vasopressors during ICU stay were also higher in septic patients. Clinical frailty at ICU admission was similar between the groups. Duration of ventilation, ICU and hospital length of stay were longer, and ICU, hospital, 90-day and 180-day mortality were higher in patients with sepsis (Fig. 1; Additional file 1: Table S7). Also, patients with sepsis were less often discharged home (Additional file 1: Table S7). These differences remained significant even after adjustment for covariates.

**Table 1:** Patient characteristics according to presence of sepsis during ICU stay

	Sepsis (*n* = 282)	No sepsis (*n* = 606)	*p* value^a^
Age, years	60.1 (47.0–69.8)	59.1 (45.4–69.6)	0.438
Male gender—no. (%)	174 (61.7)	377 (62.2)	0.882
Body mass index, kg/m^2^	27.0 (23.5–31.2)	26.6 (23.1–30.9)	0.393
Marital status—no. (%)			
Separated or divorced	7/130 (5.4)	45/353 (12.7)	0.020
Living with a loved one	76/130 (58.5)	227/353 (64.3)	0.245
APACHE III	74.0 (57.0–96.0)	59.0 (44.0–80.0)	< 0.001
Type of admission—no. (%)			< 0.001
Medical	217/280 (77.5)	321/597 (53.8)	
Surgical	63/280 (22.5)	276/597 (46.2)	
Sepsis at ICU admission	128/280 (45.7)	0/605 (0.0)	< 0.001
Acute respiratory failure	50/265 (18.9)	31/549 (5.6)	< 0.001
Cardiac arrest	13/276 (4.7)	83/583 (14.2)	< 0.001
Diagnosis category—no. (%)			< 0.001
Cardiovascular	59/280 (21.1)	238/605 (39.3)	
Gastrointestinal	24/280 (8.6)	58/605 (9.6)	
Gynecological	0/280 (0.0)	2/605 (0.3)	
Hematological	0/280 (0.0)	2/605 (0.3)	
Metabolic	3/280 (1.1)	41/605 (6.8)	
Musculoskeletal and skin	7/280 (2.5)	14/605 (2.3)	
Neurological	6/280 (2.1)	33/605 (5.5)	
Renal and genitourinary	2/280 (0.7)	6/605 (1.0)	
Respiratory	36/280 (12.9)	118/605 (19.5)	
Sepsis	128/280 (45.7)	0/605 (0.0)	
Trauma	15/280 (5.4)	93/605 (15.4)	
Co-existing disorders–—no. (%)			
Chronic respiratory failure			0.016
Lung transplant	7/279 (2.5)	41/590 (6.9)	
Other	15/279 (5.4)	24/590 (4.1)	
Chronic cardiovascular disease	12/279 (4.3)	32/590 (5.4)	0.619
Chronic liver disease	12/279 (4.3)	19/590 (3.2)	0.437
Chronic kidney disease	8/279 (2.9)	20/590 (3.4)	0.838
Chronic immune disease	8/279 (2.9)	9/590 (1.5)	0.195
Chronic immunosuppression	29/279 (10.4)	42/590 (7.1)	0.112
Diabetes	95/276 (34.4)	183/584 (31.3)	0.391
Hospital source of admission–—no. (%)			< 0.001
Home	163/276 (59.1)	408/586 (69.6)	
Other acute hospital not ICU	57/276 (20.7)	140/586 (23.9)	
Other hospital ICU	55/276 (19.9)	28/586 (4.8)	
Rehabilitation	1/276 (0.4)	6/586 (1.0)	
Nursing home	0/276 (0.0)	3/586 (0.5)	
Mental health	0/276 (0.0)	1/586 (0.2)	
ICU source of admission–—no. (%)			< 0.001
Emergency room	79/280 (28.2)	197/596 (33.1)	
Operating room	64/280 (22.9)	276/596 (46.3)	
Ward	64/280 (22.9)	63/596 (10.6)	
Other hospital ICU	70/280 (25.0)	57/596 (9.6)	
Other ICU	3/280 (1.1)	3/596 (0.5)	
Treatment limitation at ICU admission–—no. (%)	10/269 (3.7)	11/559 (2.0)	0.177
Clinical frailty score at ICU admission–—no. (%)			0.121
Non-frail	175/216 (81.0)	333/424 (78.5)	
Mild-to-moderate frail	33/216 (15.3)	84/424 (19.8)	
Severely frail	8/216 (3.7)	7/424 (1.7)	
Organ support during ICU stay–—no. (%)			
Renal replacement therapy	95/190 (50.0)	58/395 (14.7)	< 0.001
Extracorporeal membrane oxygenation	33/255 (12.9)	27/525 (5.1)	< 0.001
Noninvasive ventilation	69/238 (29.0)	83/496 (16.7)	< 0.001
Tracheostomy	22/189 (11.6)	21/402 (5.2)	0.009
Inotrope and/or vasopressor	187/193 (96.9)	333/397 (83.9)	< 0.001
Laboratory test at ICU admission			
pH	7.31 (7.24–7.40)	7.34 (7.27–7.40)	0.085
PaO_2_/FiO_2_	181.0 (112.9–288.0)	242.2 (153.1–337.4)	< 0.001
Lactate, mmol/L	2.8 (1.8–5.3)	2.4 (1.6–4.4)	0.037

Data are median (quartile 25%—quartile 75%) or No (%). Percentages may not total 100 because of rounding. Denominators are shown when the overall sample size was not available

APACHE, Acute Physiology and Chronic Health Evaluation; ICU, intensive care unit

^a^*p* values from Wilcoxon rank-sum test for continuous variables or Fisher exact test for categorical variables

### WHODAS score

Prior to ICU admission, the WHODAS score (25.7% ± 27.4 vs. 20.7% ± 24.1; mean difference, 5.00 [95% CI − 0.09 to 10.09]; *p* = 0.054) and the percentage of patients with disability (40.9% vs. 34.9%; risk difference, 6.09 [95% CI − 3.67 to 16.06]; *p* = 0.228) were numerically but not significantly greater in patients with sepsis (Additional file 1: Table S8).

### Primary outcome

At 6 months, 42/106 (39.6%) and 106/300 (35.3%) of the patients with and without sepsis reported new disability, respectively, and 142/206 (68.9%) and 228/422 (54.0%) patients with and without sepsis either reported a new disability or had died (Table 2).

**Table 2:** Long-term outcomes according to the presence of sepsis

	At 3 months^d^				At 6 months^e^				*p* interaction^b^
	Sepsis (*n* = 282)	No sepsis (*n* = 606)	Absolute difference^a^ (95% CI)	*p* value	Sepsis (*n* = 282)	No sepsis (*n* = 606)	Absolute difference^a^ (95% CI)	*p* value	
WHODAS score, %	31.8 ± 23.5	24.0 ± 22.0	MD, 3.00 (− 1.42 to 7.42)	0.184	26.1 ± 22.1	21.5 ± 21.1	MD, − 1.40 (− 6.03 to 3.23)	0.554	0.020
Disability—no. (%)	73/129 (56.6)	145/348 (41.7)	RD, 5.88 (− 4.11 to 15.90)	0.248	52/118 (44.1)	126/330 (38.2)	RD, − 2.44 (− 13.08 to 8.20)	0.653	0.111
New disability^c^—no. (%)	50/126 (39.7)	114/344 (33.1)	RD, 5.29 (− 4.45 to 15.00)	0.287	42/106 (39.6)	106/300 (35.3)	RD, 0.00 (− 10.29 to 10.40)	0.995	0.272
New disability or death—no. (%)	146/222 (65.8)	231/461 (50.1)	RD, 5.22 (− 4.52 to 15.00)	0.293	142/206 (68.9)	228/422 (54.0)	RD, 0.83 (− 9.47 to 11.10)	0.874	0.360
EuroQol-visual analogue scale	61.0 ± 24.3	67.4 ± 22.9	MD, − 3.82 (− 8.44 to 0.80)	0.105	66.1 ± 20.7	71.4 ± 20.1	MD, − 1.72 (− 6.67 to 3.23)	0.495	0.394
EQ-5D-5L™ utility	0.6 ± 0.3	0.7 ± 0.3	MD, − 0.07 (− 0.13 to − 0.01)	0.013	0.7 ± 0.3	0.7 ± 0.3	MD, − 0.04 (− 0.10 to 0.02)	0.152	0.268
No problem with anxiety	63/129 (48.8)	195/349 (55.9)	RD, − 7.60 (− 17.50 to 2.31)	0.132	71/118 (60.2)	196/332 (59.0)	RD, 1.85 (− 8.75 to 12.46)	0.732	0.079
No problem with mobility	49/129 (38.0)	204/349 (58.5)	RD, − 11.87 (− 21.80 to − 1.95)	0.019	63/118 (53.4)	220/332 (66.3)	RD, − 4.22 (− 14.90 to 6.45)	0.438	0.182
No problem with pain	51/129 (39.5)	165/349 (47.3)	RD, − 4.61 (− 15.13 to 5.91)	0.390	56/118 (47.5)	171/332 (51.5)	RD, 3.04 (− 8.23 to 14.32)	0.596	0.195
No problem with personal care	66/129 (51.2)	217/349 (62.2)	RD, − 3.84 (− 13.60 to 5.95)	0.442	64/118 (54.2)	230/332 (69.3)	RD, − 6.74 (− 17.20 to 3.75)	0.208	0.591
No problem with usual activities	34/129 (26.4)	117/349 (33.5)	RD, − 3.26 (− 13.40 to 6.87)	0.528	37/118 (31.4)	139/332 (41.9)	RD, − 1.73 (− 12.60 to 9.17)	0.755	0.791
Unemployed due to health	64/129 (49.6)	162/348 (46.6)	RD, 0.33 (− 9.82 to 10.50)	0.949	52/118 (44.1)	135/334 (40.4)	RD, − 0.17 (− 10.64 to 10.30)	0.974	0.911
IES-R	–	–	–	–	12.3 ± 14.4	8.5 ± 12.5	MD, 2.27 (− 2.09 to 6.63)	0.326	–
Post-traumatic stress disorder	–	–	–	–	8/62 (12.9)	9/184 (4.9)	RD, 7.22 (− 1.45 to 15.85)	0.116	–
IADL	–	–	–	–	6.5 ± 2.1	7.1 ± 1.7	MD, − 0.56 (− 1.00 to − 0.14)	0.013	–
Fully independent	–	–	–	–	61/117 (52.1)	214/327 (65.4)	RD, − 10.86 (− 22.67 to 0.38)	0.071	–
MoCA-BLIND	–	–	–	–	18.6 ± 2.8	18.5 ± 3.1	MD, − 0.14 (− 1.17 to 0.89)	0.803	–
Cognitive dysfunction	–	–	–	–	18/61 (29.5)	50/174 (28.7)	RD, − 0.17 (− 15.79 to 15.45)	0.983	–
HADS anxiety	–	–	–	–	4.7 ± 4.7	4.3 ± 4.3	MD, 0.81 (− 0.53 to 2.15)	0.250	–
Anxiety	–	–	–	–	19/73 (26.0)	47/212 (22.2)	RD, 3.12 (− 9.67 to 15.91)	0.644	–
HADS depression	–	–	–	–	4.1 ± 3.6	3.6 ± 3.8	MD, 0.54 (− 0.61 to 1.71)	0.378	–
Depression	–	–	–	–	15/73 (20.5)	36/208 (17.3)	RD, 7.65 (− 4.53 to 19.84)	0.234	–
Financial distress	2.7 ± 3.4	2.4 ± 3.3	MD, 0.11 (− 0.63 to 0.86)	0.766	2.2 ± 3.3	1.8 ± 3.0	MD, 0.02 (− 0.77 to 0.82)	0.954	0.816

Data are mean ± standard deviation or No (%). Percentages may not total 100 because of rounding. Denominators are shown when the overall sample size was not available

MD, mean difference; RD, risk difference

^a^All models are mixed-effect models considering the moment of measurement, group, as well as the group x time interaction as fixed effect. Moment of measurement was treated as a categorical variable, and random intercepts for patients and centers were included to account for the dependency of repeated measures and clustering of the data. Between-group comparisons at each time point was estimate with the appropriate contrasts from the model and using a Holm–Bonferroni method to adjust for multiplicity. All models were adjusted by age, sex, ICU admission source, APACHE III score, type of admission (medical vs. surgical), lung transplant patients, trauma, creatinine, heart rate, mean arterial pressure, presence of chronic cardiovascular disease and ICU length of stay. Whenever available, models were further adjusted by the baseline value of the outcome of interest as fixed effect. In all models, the no sepsis group was used as reference (OR > 1 represents increased risk is septic patients, and MD > 1 represents increase in the score in septic patients)

^b^*p* value for interaction between sepsis group and moment of measurement

^c^New disability defined as a change of WHODAS ≥ 10%

^d^675 patients were alive at 3 months (186 in the sepsis and 489 in the no sepsis group)

^e^666 patients were alive at 3 months (182 in the sepsis and 484 in the no sepsis group)

**Fig. 2:**
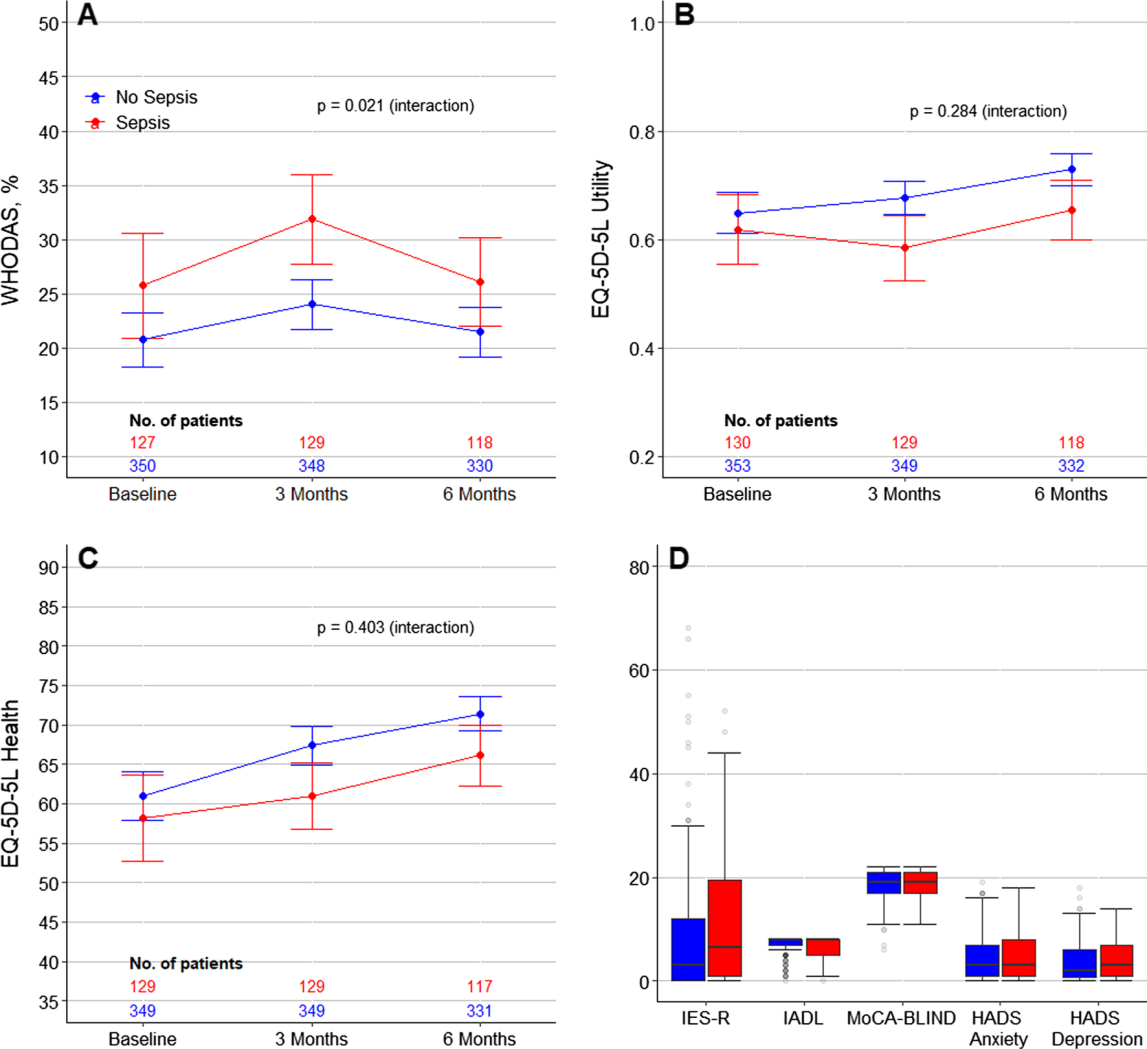
Trajectory of outcomes to 6 months in patients with sepsis (red) and without sepsis (blue). **A**–**C** Circles are mean and error bars are 95% confidence interval. *P* values calculated from the interaction between sepsis and time from a mixed-effect generalized linear model with Gaussian distribution, including center as random effect, and adjusted by age, sex, ICU admission source, APACHE III score, type of admission (medical vs. surgical), lung transplant patients, trauma, creatinine, heart rate, mean arterial pressure, presence of chronic cardiovascular disease and ICU length of stay. Models were further adjusted by the baseline value of the outcome of interest as fixed effect. **D** Outcomes assessed at 6 months of follow-up. Boxes represent median and interquartile range. Whiskers extend 1.5 times the interquartile range beyond the first and third quartiles per the conventional Tukey method. Transparent circles beyond the whiskers represent outliers. Abbreviations: WHODAS, WHO Disability Assessment Schedule 2.0; IES-R, Impact of Event Scale–Revised; IADL, instrumental activities of daily living; and MoCA-BLIND, Montreal Cognitive Assessment

**Fig. 3:**
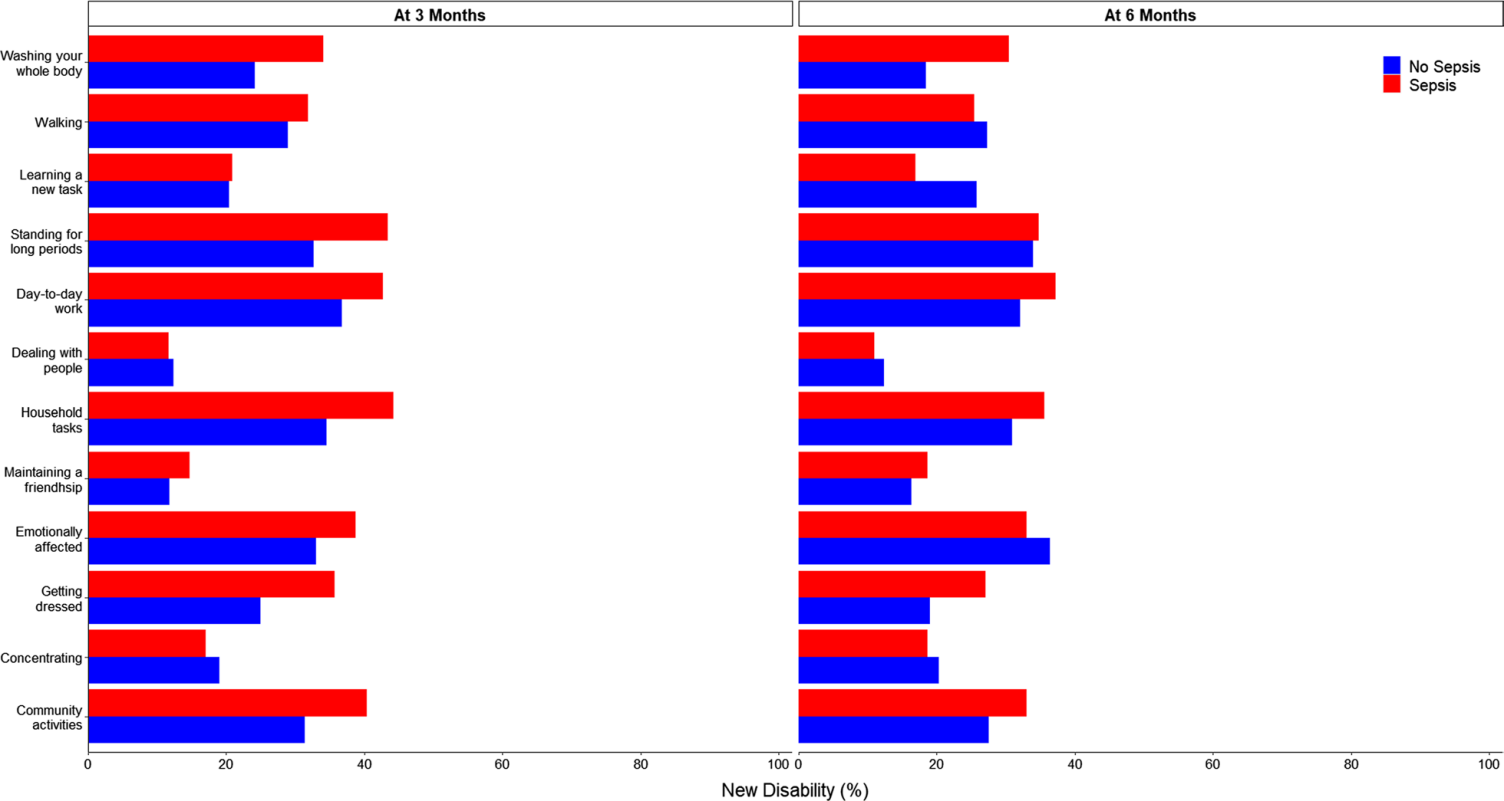
New disability at 3 and 6 months in patients with sepsis (red) and without sepsis (blue)

On unadjusted analysis, patients with sepsis had a higher WHODAS score, and an increased risk of disability at 3 months but not at 6 months (Fig. 2; Additional file 1: Figures S2 and S3 and Table S9). Development of new disability in each WHODAS score component at 6 months in patients with and without sepsis is shown in Fig. 3. The risk of new disability was similar between the groups at 3 and 6 months. However, on unadjusted analysis, the risk of new disability or death was higher in septic patients at 3 months (risk difference, 15.66 [95% CI 7.82–23.51]; *p* < 0.001) and at 6 months (risk difference, 12.92 [95% CI 4.93–20.92]; *p* = 0.002) (Additional file 1: Table S9).

After adjustment for covariates, there was no difference in WHODAS score, or risk for disability, new disability or new disability or death between the groups at 3 or 6 months (Table 2). Disability was common in all areas of functioning (e.g., standing for long periods, learning a new task, emotionally affected) (Fig. 3), and these findings were consistent after multiple imputation (Additional file 1: Table S10).

#### EQ-5D-5L

At baseline, there was no difference in the components of EQ-5D-5L between the groups (Additional file 1: Table S8). On unadjusted analysis, while EQ-VAS and utility scores were lower in patients with sepsis at 3 months, none of the scores were different between the groups at 6 months (Fig. 2; Additional file 1: Figure S2 and Table S9). In addition, a greater proportion of patients with sepsis had problems with their mobility at 3 months, and with their personal care at 6 months (Additional file 1: Figure S3 and Table S9). After adjustment for covariates, however, there was no statistically significant difference in any of the components of EQ-5D-5L between the groups at 6 months (Table 2). These findings were consistent after multiple imputation (Additional file 1: Table S10).

### Anxiety and depression

Both HADS anxiety and depression at 6 months were similar between the groups before and after adjustment (Fig. 2; Table 2; Additional file 1: Figures S2 and S3 and Table S9). These findings were consistent after multiple imputation (Additional file 1: Table S10).

### Post-traumatic stress disorder

IES-R and the risk of post-traumatic stress disorder at 6 months were similar between the groups in the unadjusted and adjusted analysis (Fig. 2; Table 2; Additional file 1: Figures S2 and S3 and Table S9). These findings were consistent after multiple imputation (Additional file 1: Table S10, Figure S4).

### Return to work

The risk of being unemployed due to health problems at 3 and 6 months was similar between the groups before and after adjustment (Table 2; Additional file 1: Table S9 and Figure S3). These findings were consistent after multiple imputation (Additional file 1: Table S10).

### Daily activities

The total IADL score, but not the proportion of patients who were fully independent at 6 months, was lower in patients with sepsis in the unadjusted analysis but not in the adjusted analysis (Fig. 2; Additional file 1: Table S9, and Figures S2 and S3). These findings were consistent after multiple imputation (Additional file 1: Table S10, Figure S4).

### Cognitive function

The proportion of patients with of cognitive dysfunction at 6 months were similar between the groups before and after adjustment (Fig. 2; Table 2; Additional file 1: Figs. 2 and 3 and Table S9). These findings were consistent after multiple imputation (Additional file 1: Table S10).

### Financial distress

Financial distress at 6 months was similar between the groups before and after adjustment (Table 2; Additional file 1: Table S9). These findings were consistent after multiple imputation (Additional file 1: Table S10).

## Discussion

In this multicenter cohort study, mechanically ventilated patients with sepsis admitted to ICU had an increased risk of death, but survivors did not have an increased risk of new disability at 6 months compared to survivors of critical illness without sepsis of similar acuity and length of stay. Among survivors, there was no difference in the incidence of new disability, the number of patients with disability or the severity of disability at 6 months according to the presence or absence of sepsis. There were also no differences between survivors with and without sepsis in health-related quality of life, anxiety and depression, post-traumatic stress disorder, return to work, financial distress or cognitive function at 6 months. An additional important finding was that, prior to the ICU admission, baseline rates of disability were high, which was associated with 6-month disability.

Despite the recognition of the importance of the quality of recovery after sepsis, very limited data exist about the prevalence of new disability in survivors of sepsis after critical illness. In one prospective cohort study of older people, 516 versus 4517 sepsis versus non-sepsis survivors of hospitalization were assessed. Their mean age was 76.9 years, and 17 years older than our cohort and most were not critically ill [5]. Survivors of severe sepsis appeared at greater risk of additional functional limitations and greater odds of moderate-to-severe cognitive impairment than their pre-illness trajectory or their hospitalization. However, this study did not focus on ICU patients and excluded non-sepsis ICU patients. In contrast, in the current study we focused on a much younger cohort. We found no difference in functional status between ICU survivors who received mechanical ventilation with and without sepsis at 6 months, providing among the first direct comparisons with information on illness severity and immediate pre-hospital disability. In our study, survivors who had sepsis had increased disability at 3 months but not at 6 months, indicating that patients with sepsis may take longer to recover from critical illness.

Our findings support the results of a recent propensity matched study of survivors with and without sepsis enrolled in a large, multicenter randomized trial [17]. After matching, there were no significant differences in the proportion of survivors with and without sepsis reporting problems with mobility, self-care, usual activities, pain/discomfort and anxiety/depression. This study did not report baseline health status and could not assess for the presence of new disability. Our study found no difference in new disability and no difference between any of the domains of the EQ-5D-5L™, the utility score and the visual analogue scale (EQ-VAS) for patients with and without sepsis.

We used the measurement of new disability relative to baseline disability, adjusted for illness severity which has previously been reported in studies of critically ill patients [6, 7]. However, we acknowledge that this may introduce recall bias that may have affected both sepsis and non-sepsis patients and this method of measurement needs further validation. We included a multicenter, heterogeneous group of critically ill patients to increase generalizability [19]. The functional outcome measures were comprehensive and included validated tools recommended for survivors of acute respiratory failure [20]. We used trained, blinded outcome assessors who were located centrally to allow for monitoring.

We acknowledge several limitations. A proportion of patients were lost to follow-up, although it was a much lower proportion compared to previous studies of sepsis survivors. The responders were similar to the non-responders, and therefore, the results likely represent the overall cohort of eligible patients. The definition of sepsis was pragmatic, using APACHE III and hospital ICD 10 AM coding (A41), which risks misclassification, and the results may have been different if we had used the Sepsis 3 definition. Although we adjusted for baseline imbalances, including for diagnoses, there were differences between the APACHE III diagnoses for the sepsis and non-sepsis groups that may be important. In person objective measurements of function were not possible. We were unable to report hospital readmissions, and subsequent illnesses may influence long-term disability.

## Conclusion

Compared to mechanically ventilated patients of similar acuity and length of stay without sepsis, patients with sepsis admitted to ICU have an increased risk of death, but survivors have a similar risk of new disability at 6 months.

## Supplementary Information

**Additional file 1. **The electronic supplement includes additional information about the study including methods and results, participating sites, missing data, adjusted and unadjusted analyses, the flowchart of included participants, trajectory of long-term outcomes and incidence of disability.

## Data Availability

Partial data set sharing is according to individual requests for data access. Requests will be considered by the study's Management Committee. Requests for data sharing are to be made to anzicrc@monash.edu and the corresponding author, carol.hodgson@monash.edu.

## Code availability

Statistical code is available on request to anzicrc@monash.edu and the corresponding author, carol.hodgson@monash.edu.
