# Supplementary material for: Comparison of 6-month outcomes of sepsis versus non-sepsis critically ill patients receiving mechanical ventilation
Source: Crit Care. 2022 Jun 13;26:174. doi: 10.1186/s13054-022-04041-w (PMC9189265; doi:10.1186/s13054-022-04041-w)
Supplement: Supplementary file 1 — Additional file 1. The electronic supplement includes additional information about the study including methods and results, participating sites, missing data, adjusted and unadjusted analyses, the flowchart of included participants, trajectory of long-term outcomes and incidence of disability. [file 13054_2022_4041_MOESM1_ESM.docx]

**The Association Between Sepsis And New Disability In Critically Ill Patients Receiving Mechanical Ventilation**

ONLINE SUPPLEMENT

The PREDICT Study Investigators

**Writing committee:** Carol L.Hodgson, PhD, FACP, BAppSc(PT);^1,2^ Alisa M. Higgins, PhD, MPH, GDipBiostat, BPhysio(Hons);^1^ Michael Bailey, PhD, MSc, BSc(Hons);^1^ Jonathon Barrett, MBBS, FRACP, FCICM, MPH;^3,4^ Rinaldo Bellomo, AO, MBBS, MD, PhD, FRACP, FCICM, FAMHS;^1,5,6^ D. James Cooper, AO, BMBS, MD, FRACP, FCICM, FAHMS;^1,2^ Belinda J. Gabbe, PhD, MBiostat, MAppSc;^7^ Theodore Iwashyna, MD PhD^8,^; Natalie Linke, BN GCert(IntCareNurs) MN;^1^ Paul S. Myles, MBBS, MPH, DSc, FCAI, FANZCA, FRCA;^9^ Michelle Paton MPhty(Cardio), MPhty, BExSci;^1,10^ Steve Philpot, MBBS(Hons), FANZCA, FCICM;^11^ Mark Shulman, MBBS, MPH, FANZCA;^9^ Meredith Young MPH BNurs GradCertNur(IntC) RN;^2^ Ary Serpa Neto, PhD MSc MD^1,5,6,12^

Contents

[eMETHODS 3](#_Toc100652692)

[eTable 1 - Participating Sites 5](#_Toc100652693)

[eTable 2 - Amount of Missing Data in the Groups Assessed 6](#_Toc100652694)

[eTable 3 - Amount of Missing Data Assessed For Survivors at 6 Months 7](#_Toc100652695)

[eTable 4 - Patient Characteristics According to the Different Cohorts 9](#_Toc100652696)

[eTable 5 - Patient Characteristics According to Respondents and Non-Respondents at 6 Months 11](#_Toc100652697)

[eTable 6 - Clinical Outcomes According to Presence of Sepsis During ICU Stay 15](#_Toc100652698)

[eTable 7 - Baseline Function and Health Status According to the Presence of Sepsis* 16](#_Toc100652699)

[eTable 8 - Long-Term Outcomes According to the Presence of Sepsis (Unadjusted Analyses) 17](#_Toc100652700)

[eTable 9 - Long-Term Outcomes According to the Presence of Sepsis After Multiple Imputation 18](#_Toc100652701)

[eTable 10 – APACHE III Diagnoses of Patients without Sepsis 19](#_Toc100652702)

[eFigure 1 - Flowchart of Inclusion 29](#_Toc100652703)

[eFigure 2 - Trajectory of Long-Term Outcomes in Patients with and Without Sepsis 30](#_Toc100652704)

[eFigure 3 - Incidence of Disability, Problems Related to the EQ-5D-5L, Unemployment due to Health Problems at Baseline and 3 Months (an increase in disability and unemployed indicates a worse outcome, an increase in “no problem” indicates a better outcome) 31](#_Toc100652705)

[eFigure 4 - Incidence of Disability, Problems Related to the EQ-5D-5L, Unemployment due to Health Problems, Post-Traumatic Stress Disorder, Independence and Cognitive Dysfunction at 6 Months(an increase in disability, cognitive dysfunction and unemployed indicates a worse outcome, an increase in “no problem” indicates a better outcome) 32](#_Toc100652706)

# eMETHODS

*Multiple imputation*

For patients with missing values in long-term outcomes, multiple imputation by chained equation method (MICE) was used. By leveraging known patient characteristics and accounting for uncertainty in the multiple estimations of missing values, multiple imputation preserves sample size and reduces bias while examining association between variables. Before imputation, the percentage of missingness in the variables was assessed.

The following variables were considered as predictors in the multiple imputation model: age, gender, presence of sepsis, APACHE III, body mass index, clinical frailty, baseline score of interest, type of admission (medical or surgical), admission diagnosis, ICU source of admission, mental and behavioral disorders, chronic respiratory disease (including lung transplant indicator), chronic cardiovascular disease, chronic immunosuppression, diabetes, malnutrition, ARDS, score of interest at 3 and 6 months, ICU length of stay, and the following variables assessed in the first 24 hours of ICU admission: highest temperature, highest respiratory rate, highest white blood cell count, highest platelets, highest potassium, pH, PaO_2_ / FiO_2_ ratio, lactate, highest creatinine, lowest mean arterial pressure and highest heart rate.

The lower and upper cut-offs of imputed values respected the upper and lower limits of the original variables in patients without missing data. Imputation was performed using predictive mean matching for continuous variables, logistic regression for binary variables and polytomous regression for categorical variables with more than two categories. A model in five imputed datasets with 50 iterations was used. After imputation all models were repeated and results combined across imputations using Rubin’s rule.

| eTable 1 - Participating Sites | | | | |
| --- | --- | --- | --- | --- |
| **Participating Site** | **Hospital Type** | **Total number of available ICU beds** | **No. patients recruited**  **Hospital Cohort** | **No. patients recruited**  **Follow-Up Cohort** |
| Austin Hospital | Public | 21 | 115 | 90 |
| Alfred Hospital | Public | 38 | 523 | 404 |
| Cabrini Hospital | Private | 16 | 10 | 9 |
| Dandenong Hospital | Public | 14 | 77 | 43 |
| Epworth Hospital | Private | 26 | 39 | 37 |
| Monash Medical Centre | Public | 25 | 124 | 87 |

| eTable 2 - Amount of Missing Data in the Groups Assessed | | | |
| --- | --- | --- | --- |
|  | **All Patients**  **(*n* = 888)** | **Sepsis**  **(*n* = 282)** | **No Sepsis**  **(*n* = 606)** |
| Age | 0 (0.0) | 0 (0.0) | 0 (0.0) |
| Sex | 0 (0.0) | 0 (0.0) | 0 (0.0) |
| Body mass index | 233 (26.2) | 54 (19.1) | 179 (29.5) |
| Marital status | 405 (45.6) | 152 (53.9) | 253 (41.7) |
| APACHE III | 17 (1.9) | 3 (1.1) | 14 (2.3) |
| Type of admission | 11 (1.2) | 2 (0.7) | 9 (1.5) |
| Sepsis at baseline | 3 (0.3) | 2 (0.7) | 1 (0.2) |
| Acute respiratory failure at baseline | 74 (8.3) | 17 (6.0) | 57 (9.4) |
| Cardiac arrest at baseline | 29 (3.3) | 6 (2.1) | 23 (3.8) |
| Admission diagnosis | 3 (0.3) | 2 (0.7) | 1 (0.2) |
| Chronic lung disease | 19 (2.1) | 3 (1.1) | 16 (2.6) |
| Chronic cardiovascular disease | 19 (2.1) | 3 (1.1) | 16 (2.6) |
| Chronic liver disease | 19 (2.1) | 3 (1.1) | 16 (2.6) |
| Chronic kidney disease | 19 (2.1) | 3 (1.1) | 16 (2.6) |
| Chronic immune disease | 19 (2.1) | 3 (1.1) | 16 (2.6) |
| Chronic immunosuppression | 19 (2.1) | 3 (1.1) | 16 (2.6) |
| Diabetes | 28 (3.2) | 6 (2.1) | 22 (3.6) |
| Hospital source of admission | 26 (2.9) | 6 (2.1) | 20 (3.3) |
| ICU source of admission | 12 (1.4) | 2 (0.7) | 10 (1.7) |
| Treatment limitation at ICU admission | 60 (6.8) | 13 (4.6) | 47 (7.8) |
| Clinical frailty score at ICU admission | 248 (27.9) | 66 (23.4) | 182 (30.0) |
| Renal replacement therapy during ICU stay | 303 (34.1) | 92 (32.6) | 211 (34.8) |
| Extracorporeal membrane oxygenation during ICU stay | 108 (12.2) | 27 (9.6) | 81 (13.4) |
| Non-invasive ventilation during ICU stay | 154 (17.3) | 44 (15.6) | 110 (18.2) |
| Tracheostomy during ICU stay | 297 (33.4) | 93 (33.0) | 204 (33.7) |
| Inotrope and/or vasopressor during ICU stay | 298 (33.6) | 89 (31.6) | 209 (34.5) |
| pH at ICU admission | 91 (10.2) | 25 (8.9) | 66 (10.9) |
| PaO_2_ / FiO_2_ at ICU admission | 91 (10.2) | 25 (8.9) | 66 (10.9) |
| Lactate at ICU admission | 132 (14.9) | 37 (13.1) | 95 (15.7) |
| This data includes patients who died before 6-months | | | |

| eTable 3 - Amount of Missing Data Assessed For Survivors at 6 Months | | | |
| --- | --- | --- | --- |
|  | **All Patients**  **(*n* = 666)** | **Sepsis**  **(*n* = 182)** | **No Sepsis**  **(*n* = 484)** |
| Age | 0 (0.0) | 0 (0.0) | 0 (0.0) |
| Sex | 0 (0.0) | 0 (0.0) | 0 (0.0) |
| Body mass index | 173 (26.0) | 36 (19.8) | 137 (28.3) |
| Marital status | 185 (27.8) | 53 (29.1) | 132 (27.3) |
| APACHE III | 16 (2.4) | 3 (1.6) | 13 (2.7) |
| Type of admission | 10 (1.5) | 2 (1.1) | 8 (1.7) |
| Sepsis at baseline | 2 (0.3) | 2 (1.1) | 0 (0.0) |
| Acute respiratory failure at baseline | 57 (8.6) | 13 (7.1) | 44 (9.1) |
| Cardiac arrest at baseline | 14 (2.1) | 2 (1.1) | 12 (2.5) |
| Admission diagnosis | 2 (0.3) | 2 (1.1) | 0 (0.0) |
| Chronic lung disease | 18 (2.7) | 3 (1.6) | 15 (3.1) |
| Chronic cardiovascular disease | 18 (2.7) | 3 (1.6) | 15 (3.1) |
| Chronic liver disease | 18 (2.7) | 3 (1.6) | 15 (3.1) |
| Chronic kidney disease | 18 (2.7) | 3 (1.6) | 15 (3.1) |
| Chronic immune disease | 18 (2.7) | 3 (1.6) | 15 (3.1) |
| Chronic immunosuppression | 18 (2.7) | 3 (1.6) | 15 (3.1) |
| Diabetes | 12 (1.8) | 2 (1.1) | 10 (2.1) |
| Hospital source of admission | 12 (1.8) | 2 (1.1) | 10 (2.1) |
| ICU source of admission | 11 (1.7) | 2 (1.1) | 9 (1.9) |
| Treatment limitation at ICU admission | 45 (6.8) | 9 (4.9) | 36 (7.4) |
| Clinical frailty score at ICU admission | 178 (26.7) | 37 (20.3) | 141 (29.1) |
| Renal replacement therapy during ICU stay | 244 (36.6) | 63 (34.6) | 181 (37.4) |
| Extracorporeal membrane oxygenation during ICU stay | 87 (13.1) | 20 (11.0) | 67 (13.8) |
| Non-invasive ventilation during ICU stay | 120 (18.0) | 28 (15.4) | 92 (19.0) |
| Tracheostomy during ICU stay | 237 (35.6) | 63 (34.6) | 174 (36.0) |
| Inotrope and/or vasopressor during ICU stay | 240 (36.0) | 60 (33.0) | 180 (37.2) |
| pH at ICU admission | 67 (10.1) | 16 (8.8) | 51 (10.5) |
| PaO_2_ / FiO_2_ at ICU admission | 67 (10.1) | 16 (8.8) | 51 (10.5) |
| Lactate at ICU admission | 104 (15.6) | 25 (13.7) | 79 (16.3) |
| WHODAS at baseline | 191 (28.7) | 56 (30.8) | 135 (27.9) |
| WHODAS at 3 months | 191 (28.7) | 54 (29.7) | 137 (28.3) |
| WHODAS at 6 months | 218 (32.7) | 64 (35.2) | 154 (31.8) |
| EQ-5D-5L health at baseline | 190 (28.5) | 54 (29.7) | 136 (28.1) |
| EQ-5D-5L health at 3 months | 190 (28.5) | 54 (29.7) | 136 (28.1) |
| EQ-5D-5L health at 6 months | 218 (32.7) | 65 (35.7) | 153 (31.6) |
| EQ-5D-5L utility at baseline | 185 (27.8) | 53 (29.1) | 132 (27.3) |
| EQ-5D-5L utility at 3 months | 190 (28.5) | 54 (29.7) | 136 (28.1) |
| EQ-5D-5L utility at 6 months | 216 (32.4) | 64 (35.2) | 152 (31.4) |
| EQ-5D-5L mobility at baseline | 185 (27.8) | 53 (29.1) | 132 (27.3) |
| EQ-5D-5L personal care at baseline | 185 (27.8) | 53 (29.1) | 132 (27.3) |
| EQ-5D-5L usual activities at baseline | 185 (27.8) | 53 (29.1) | 132 (27.3) |
| EQ-5D-5L pain at baseline | 185 (27.8) | 53 (29.1) | 132 (27.3) |
| EQ-5D-5L anxiety and depression at baseline | 185 (27.8) | 53 (29.1) | 132 (27.3) |
| EQ-5D-5L mobility at 3 months | 190 (28.5) | 54 (29.7) | 136 (28.1) |
| EQ-5D-5L personal care at 3 months | 190 (28.5) | 54 (29.7) | 136 (28.1) |
| EQ-5D-5L usual activities at 3 months | 190 (28.5) | 54 (29.7) | 136 (28.1) |
| EQ-5D-5L pain at 3 months | 190 (28.5) | 54 (29.7) | 136 (28.1) |
| EQ-5D-5L anxiety and depression at 3 months | 190 (28.5) | 54 (29.7) | 136 (28.1) |
| EQ-5D-5L mobility at 6 months | 216 (32.4) | 64 (35.2) | 152 (31.4) |
| EQ-5D-5L personal care at 6 months | 216 (32.4) | 64 (35.2) | 152 (31.4) |
| EQ-5D-5L usual activities at 6 months | 216 (32.4) | 64 (35.2) | 152 (31.4) |
| EQ-5D-5L pain at 6 months | 216 (32.4) | 64 (35.2) | 152 (31.4) |
| EQ-5D-5L anxiety and depression at 6 months | 216 (32.4) | 64 (35.2) | 152 (31.4) |
| HADS anxiety at 6 months | 381 (57.2) | 109 (59.9) | 272 (56.2) |
| HADS depression at 6 months | 385 (57.8) | 109 (59.9) | 276 (57.0) |
| Unemployed due to health at 3 months | 191 (28.7) | 54 (29.7) | 137 (28.3) |
| Unemployed due to health at 6 months | 214 (32.1) | 64 (35.2) | 150 (31.0) |
| IES-R at 6 months | 420 (63.1) | 120 (65.9) | 300 (62.0) |
| IADL at 6 months | 222 (33.3) | 65 (35.7) | 157 (32.4) |
| MoCA-BLIND at 6 months | 431 (64.7) | 121 (66.5) | 310 (64.0) |
| Financial distress at 3 months | 228 (34.2) | 66 (36.3) | 162 (33.5) |
| Financial distress at 6 months | 287 (43.1) | 86 (47.3) | 201 (41.5) |
| Duration of ventilation | 0 (0.0) | 0 (0.0) | 0 (0.0) |
| ICU length of stay | 0 (0.0) | 0 (0.0) | 0 (0.0) |
| Hospital length of stay | 0 (0.0) | 0 (0.0) | 0 (0.0) |
| Discharged home | 16 (2.4) | 3 (1.6) | 13 (2.7) |
| ICU mortality | 0 (0.0) | 0 (0.0) | 0 (0.0) |
| Hospital mortality | 0 (0.0) | 0 (0.0) | 0 (0.0) |
| 90-day mortality | 0 (0.0) | 0 (0.0) | 0 (0.0) |
| 180-day mortality | 0 (0.0) | 0 (0.0) | 0 (0.0) |

# eTable 4 – APACHE III Diagnoses of Patients without Sepsis

|  | **No Sepsis**  **(*n* = 606)** |
| --- | --- |
| APACHE III Subcode |  |
| 101.01 | 12 (2.0) |
| 101.02 | 1 (0.2) |
| 102 | 4 (0.7) |
| 102.01 | 71 (11.9) |
| 102.02 | 1 (0.2) |
| 103.01 | 3 (0.5) |
| 104.01 | 2 (0.3) |
| 106.04 | 1 (0.2) |
| 107.02 | 3 (0.5) |
| 107.03 | 4 (0.7) |
| 107.05 | 1 (0.2) |
| 109.03 | 2 (0.3) |
| 109.08 | 1 (0.2) |
| 110.01 | 1 (0.2) |
| 201.01 | 3 (0.5) |
| 203 | 1 (0.2) |
| 203.01 | 3 (0.5) |
| 204.01 | 3 (0.5) |
| 206 | 1 (0.2) |
| 206.01 | 6 (1.0) |
| 207.01 | 2 (0.3) |
| 208.01 | 4 (0.7) |
| 209.01 | 4 (0.7) |
| 210.01 | 1 (0.2) |
| 210.02 | 1 (0.2) |
| 211 | 1 (0.2) |
| 211.02 | 2 (0.3) |
| 211.06 | 2 (0.3) |
| 211.09 | 3 (0.5) |
| 211.1 | 1 (0.2) |
| 211.11 | 2 (0.3) |
| 212 | 2 (0.3) |
| 212.01 | 14 (2.3) |
| 212.02 | 4 (0.7) |
| 213.01 | 1 (0.2) |
| 301 | 1 (0.2) |
| 301.01 | 2 (0.3) |
| 301.02 | 3 (0.5) |
| 305 | 1 (0.2) |
| 305.01 | 1 (0.2) |
| 305.02 | 3 (0.5) |
| 306.01 | 2 (0.3) |
| 307.01 | 1 (0.2) |
| 309 | 1 (0.2) |
| 309.01 | 1 (0.2) |
| 310.01 | 1 (0.2) |
| 311 | 1 (0.2) |
| 404.02 | 2 (0.3) |
| 404.03 | 1 (0.2) |
| 405.01 | 1 (0.2) |
| 406.02 | 1 (0.2) |
| 406.04 | 2 (0.3) |
| 407.01 | 12 (2.0) |
| 408 | 1 (0.2) |
| 410.01 | 5 (0.8) |
| 601.05 | 1 (0.2) |
| 601.06 | 4 (0.7) |
| 601.08 | 1 (0.2) |
| 602.03 | 1 (0.2) |
| 602.06 | 3 (0.5) |
| 602.07 | 2 (0.3) |
| 602.08 | 1 (0.2) |
| 602.09 | 4 (0.7) |
| 602.1 | 2 (0.3) |
| 602.11 | 3 (0.5) |
| 602.13 | 1 (0.2) |
| 602.14 | 1 (0.2) |
| 602.15 | 1 (0.2) |
| 602.19 | 1 (0.2) |
| 602.2 | 2 (0.3) |
| 602.21 | 1 (0.2) |
| 603.01 | 13 (2.2) |
| 604.02 | 3 (0.5) |
| 604.03 | 1 (0.2) |
| 604.05 | 1 (0.2) |
| 604.06 | 10 (1.7) |
| 605.01 | 3 (0.5) |
| 701.01 | 1 (0.2) |
| 702.01 | 2 (0.3) |
| 703 | 2 (0.3) |
| 703.03 | 2 (0.3) |
| 703.04 | 3 (0.5) |
| 703.05 | 2 (0.3) |
| 703.06 | 2 (0.3) |
| 703.07 | 5 (0.8) |
| 703.08 | 7 (1.2) |
| 703.09 | 9 (1.5) |
| 704.01 | 6 (1.0) |
| 802.03 | 1 (0.2) |
| 802.1 | 1 (0.2) |
| 901.03 | 3 (0.5) |
| 903.01 | 1 (0.2) |
| 1102 | 1 (0.2) |
| 1102.01 | 2 (0.3) |
| 1202.03 | 1 (0.2) |
| 1204.01 | 2 (0.3) |
| 1204.02 | 1 (0.2) |
| 1205.01 | 1 (0.2) |
| 1206 | 3 (0.5) |
| 1206.01 | 5 (0.8) |
| 1206.05 | 1 (0.2) |
| 1206.06 | 2 (0.3) |
| 1206.07 | 2 (0.3) |
| 1206.08 | 4 (0.7) |
| 1206.1 | 1 (0.2) |
| 1207 | 10 (1.7) |
| 1207.01 | 32 (5.4) |
| 1207.03 | 4 (0.7) |
| 1208 | 1 (0.2) |
| 1208.02 | 1 (0.2) |
| 1208.03 | 1 (0.2) |
| 1208.06 | 12 (2.0) |
| 1208.09 | 1 (0.2) |
| 1208.1 | 2 (0.3) |
| 1208.22 | 8 (1.3) |
| 1208.24 | 1 (0.2) |
| 1209 | 2 (0.3) |
| 1209.01 | 3 (0.5) |
| 1209.02 | 7 (1.2) |
| 1210.01 | 1 (0.2) |
| 1210.02 | 1 (0.2) |
| 1212 | 5 (0.8) |
| 1212.01 | 1 (0.2) |
| 1212.02 | 1 (0.2) |
| 1212.03 | 2 (0.3) |
| 1212.04 | 4 (0.7) |
| 1212.05 | 4 (0.7) |
| 1213.01 | 1 (0.2) |
| 1301 | 1 (0.2) |
| 1301.01 | 1 (0.2) |
| 1302.02 | 2 (0.3) |
| 1303 | 2 (0.3) |
| 1303.01 | 2 (0.3) |
| 1303.02 | 1 (0.2) |
| 1304 | 1 (0.2) |
| 1304.04 | 1 (0.2) |
| 1304.05 | 3 (0.5) |
| 1304.1 | 1 (0.2) |
| 1304.11 | 40 (6.7) |
| 1401 | 4 (0.7) |
| 1401.01 | 2 (0.3) |
| 1403 | 1 (0.2) |
| 1403.01 | 1 (0.2) |
| 1403.03 | 4 (0.7) |
| 1403.04 | 4 (0.7) |
| 1404 | 1 (0.2) |
| 1404.01 | 3 (0.5) |
| 1405 | 1 (0.2) |
| 1405.04 | 2 (0.3) |
| 1405.07 | 1 (0.2) |
| 1406.01 | 1 (0.2) |
| 1407.01 | 5 (0.8) |
| 1408 | 1 (0.2) |
| 1410 | 1 (0.2) |
| 1410.01 | 4 (0.7) |
| 1412.01 | 2 (0.3) |
| 1501.01 | 1 (0.2) |
| 1502.02 | 1 (0.2) |
| 1503.01 | 1 (0.2) |
| 1504 | 1 (0.2) |
| 1504.02 | 1 (0.2) |
| 1504.03 | 2 (0.3) |
| 1602.01 | 1 (0.2) |
| 1602.04 | 1 (0.2) |
| 1602.07 | 1 (0.2) |
| 1602.09 | 2 (0.3) |
| 1602.1 | 2 (0.3) |
| 1602.11 | 2 (0.3) |
| 1602.12 | 1 (0.2) |
| 1602.14 | 2 (0.3) |
| 1602.15 | 1 (0.2) |
| 1602.17 | 1 (0.2) |
| 1602.21 | 1 (0.2) |
| 1603.01 | 8 (1.3) |
| 1604.01 | 1 (0.2) |
| 1604.04 | 1 (0.2) |
| 1604.05 | 6 (1.0) |
| 1605.01 | 2 (0.3) |
| 1701.01 | 1 (0.2) |
| 1704.01 | 1 (0.2) |
| 1802 | 1 (0.2) |
| 1803.02 | 1 (0.2) |
| 1902.04 | 1 (0.2) |
| 1902.05 | 1 (0.2) |
| 1903 | 2 (0.3) |
| 1904.01 | 7 (1.2) |
| APACHE III description |  |
| Acute myocardial infarction | 8 (1.3) |
| Aortic aneurysm | 3 (0.5) |
| Aspiration pneumonia | 3 (0.5) |
| Asthma | 4 (0.7) |
| Bacterial pneumonia | 20 (3.4) |
| Burns | 21 (3.5) |
| CABG with valve repair/replacement | 17 (2.8) |
| Cardiac arrest | 76 (12.7) |
| Cardiogenic shock | 13 (2.2) |
| Cardiomyopathy | 1 (0.2) |
| Carotid endarterectomy | 1 (0.2) |
| Cellulitis/ soft tissue infection | 10 (1.7) |
| Cholecystitis/ cholangitis | 1 (0.2) |
| Chronic obstructive pulmonary disease | 7 (1.2) |
| Coma | 5 (0.8) |
| Congestive heart failure | 2 (0.3) |
| Coronary artery bypass grafts | 46 (7.7) |
| Diabetic ketoacidosis | 2 (0.3) |
| Dissecting aortic aneurysm | 12 (2.0) |
| Drug overdose | 32 (5.4) |
| Elective AAA | 3 (0.5) |
| Endoluminal aortic repair | 1 (0.2) |
| GI bleeding | 10 (1.7) |
| GI bleeding ? diverticulosis | 2 (0.3) |
| GI bleeding ? ulcer/laceration | 5 (0.8) |
| GI neoplasm | 4 (0.7) |
| GI obstruction | 6 (1.0) |
| GI perforation/ rupture | 6 (1.0) |
| GI vascular insufficiency | 1 (0.2) |
| GI vascular ischaemia resection surgery | 5 (0.8) |
| Haemorrhage post-partum | 1 (0.2) |
| Head trauma multi trauma | 6 (1.0) |
| Hepatic failure | 6 (1.0) |
| Intracerebral haemorrhage | 1 (0.2) |
| Isolated cervical spine injury | 5 (0.8) |
| Kidney transplant | 1 (0.2) |
| Laminectomy/ spinal Cord Surgery | 4 (0.7) |
| Liver transplant | 5 (0.8) |
| Mechanical airway obstruction | 4 (0.7) |
| Metabolic coma | 1 (0.2) |
| Multi trauma with spinal injury | 23 (3.9) |
| Multiple trauma excluding head | 38 (6.4) |
| Neurologic infection | 3 (0.5) |
| Neurologic neoplasm | 1 (0.2) |
| Neuromuscular disease | 3 (0.5) |
| Orthopaedic surgery | 2 (0.3) |
| Other cardiovascular disease | 3 (0.5) |
| Other cardiovascular diseases | 27 (4.5) |
| Other GI disease | 1 (0.2) |
| Other GI diseases | 1 (0.2) |
| Other gynaecological disease | 1 (0.2) |
| Other haematologic disorders | 2 (0.3) |
| Other metabolic disorders | 6 (1.0) |
| Other neurologic disease | 1 (0.2) |
| Other respiratory diseases | 57 (9.5) |
| Pancreatitis | 1 (0.2) |
| Parasitic pneumonia | 2 (0.3) |
| Peripheral vascular disease ? No Graft | 1 (0.2) |
| Peritonitis | 2 (0.3) |
| Pregnancy-related disorder | 1 (0.2) |
| Pulmonary embolism | 2 (0.3) |
| Pulmonary oedema ? non-cardiac | 3 (0.5) |
| Renal disorders | 3 (0.5) |
| Renal neoplasm | 1 (0.2) |
| Respiratory arrest | 4 (0.7) |
| Respiratory infection | 2 (0.3) |
| Respiratory neoplasm ? lung | 2 (0.3) |
| Respiratory neoplasm ? mouth | 5 (0.8) |
| Rhythm disturbance | 1 (0.2) |
| Ruptured aortic aneurysm | 2 (0.3) |
| Seizure | 12 (2.0) |
| Skin surgery | 2 (0.3) |
| Subarachnoid haemorrhage | 1 (0.2) |
| Subdural/ epidural haematoma | 1 (0.2) |
| Valvular heart surgery | 18 (3.0) |
| Viral pneumonia | 1 (0.2) |

| eTable 5 - Patient Characteristics According to the Different Cohorts | | | | |
| --- | --- | --- | --- | --- |
|  | **Follow-Up Cohort**  **(*n* = 448)** | | **Dead at 6 Months**  **(*n* = 222)** | |
|  | **Sepsis**  **(*n* = 118)** | **No Sepsis**  **(*n* = 330)** | **Sepsis**  **(*n* = 100)** | **No Sepsis**  **(*n* = 122)** |
| Age, years | 57.0 (42.9 - 68.2) | 58.5 (46.6 - 67.4) | 64.6 (55.3 - 71.9) | 68.6 (57.0 - 76.2) |
| Male gender – no. (%) | 76 (64.4) | 215 (65.2) | 63 (63.0) | 80 (65.6) |
| Body mass index, kg/m^2^ | 27.8 (23.9 - 33.0) | 26.8 (23.2 - 30.9) | 26.0 (23.2 - 29.2) | 26.7 (23.2 - 31.0) |
| Marital status – no. (%) |  |  |  |  |
| Separated or divorced | 6 (5.6) | 37 (12.2) | 0 (0.0) | 0 (0.0) |
| Living with a loved one | 62 (57.9) | 204 (67.1) | 1 (100.0) | 1 (100.0) |
| APACHE III | 71 (54 - 84) | 55 (43 - 70) | 84 (63 - 103.2) | 85 (64 - 109) |
| Type of admission – no. (%) |  |  |  |  |
| Medical | 89 (75.4) | 149 (46.0) | 79 (79.0) | 89 (73.6) |
| Surgical | 29 (24.6) | 175 (54.0) | 21 (21.0) | 32 (26.4) |
| Acute respiratory failure | 16 (14.0) | 12 (3.8) | 23 (24.0) | 16 (14.7) |
| Cardiac arrest | 7 (5.9) | 35 (10.8) | 4 (4.2) | 35 (31.5) |
| Diagnosis category – no. (%) |  |  |  |  |
| Cardiovascular | 26 (22.0) | 124 (37.6) | 21 (21.0) | 66 (54.5) |
| Gastrointestinal | 7 (5.9) | 25 (7.6) | 13 (13.0) | 13 (10.7) |
| Gynecological | 0 (0.0) | 1 (0.3) | 0 (0.0) | 0 (0.0) |
| Hematological | 0 (0.0) | 1 (0.3) | 0 (0.0) | 1 (0.8) |
| Metabolic | 3 (2.5) | 21 (6.4) | 0 (0.0) | 3 (2.5) |
| Musculoskeletal and skin | 2 (1.6) | 7 (2.1) | 3 (3.0) | 1 (0.8) |
| Neurological | 2 (1.7) | 17 (5.2) | 3 (3.0) | 6 (5.0) |
| Renal and genitourinary | 1 (0.8) | 4 (1.2) | 1 (1.0) | 1 (0.8) |
| Respiratory | 16 (13.6) | 73 (22.1) | 12 (12.0) | 20 (16.5) |
| Sepsis | 52 (44.1) | 0 (0.0) | 45 (45.0) | 0 (0.0) |
| Trauma | 9 (7.6) | 57 (17.3) | 2 (2.0) | 10 (8.3) |
| Co-existing disorders – no. (%) |  |  |  |  |
| Chronic respiratory failure |  |  |  |  |
| Lung transplant | 4 (3.4) | 33 (10.2) | 1 (1.0) | 1 (0.8) |
| Other | 3 (2.5) | 10 (3.1) | 10 (10.0) | 11 (9.1) |
| Chronic cardiovascular disease | 5 (4.2) | 20 (6.2) | 5 (5.0) | 4 (3.3) |
| Chronic liver disease | 4 (3.4) | 5 (1.5) | 6 (6.0) | 7 (5.8) |
| Chronic kidney disease | 2 (1.7) | 4 (1.2) | 6 (6.0) | 14 (11.6) |
| Chronic immune disease | 3 (2.5) | 1 (0.3) | 5 (5.0) | 6 (5.0) |
| Chronic immunosuppression | 7 (5.9) | 20 (6.2) | 17 (17.0) | 14 (11.6) |
| Diabetes | 48 (40.7) | 110 (33.3) | 28 (29.2) | 32 (29.1) |
| Hospital source of admission – no. (%) |  |  |  |  |
| Home | 67 (56.8) | 224 (69.3) | 58 (60.4) | 75 (67.0) |
| Other acute hospital not ICU | 26 (22.0) | 84 (26.0) | 19 (19.8) | 24 (21.4) |
| Other hospital ICU | 25 (21.2) | 14 (4.3) | 18 (18.8) | 7 (6.2) |
| Rehabilitation | 0 (0.0) | 1 (0.3) | 1 (1.0) | 3 (2.7) |
| Nursing home | 0 (0.0) | 0 (0.0) | 0 (0.0) | 3 (2.7) |
| Mental health | 0 (0.0) | 0 (0.0) | 0 (0.0) | 0 (0.0) |
| ICU source of admission – no. (%) |  |  |  |  |
| Emergency room | 33 (28.0) | 99 (30.7) | 25 (25.0) | 43 (35.5) |
| Operating room | 31 (26.3) | 173 (53.6) | 20 (20.0) | 37 (30.6) |
| Ward | 21 (17.8) | 22 (6.8) | 30 (30.0) | 26 (21.5) |
| Other hospital | 33 (27.9) | 28 (8.7) | 22 (22.0) | 14 (11.6) |
| Other ICU | 0 (0.0) | 1 (0.3) | 3 (3.0) | 1 (0.8) |
| Treatment limitation at ICU admission – no. (%) | 1 (0.9) | 0 (0.0) | 7 (7.3) | 10 (9.0) |
| Clinical frailty score at ICU admission – no. (%) |  |  |  |  |
| Non-frail | 84 (88.4) | 200 (81.0) | 49 (69.0) | 57 (70.4) |
| Mild-to-moderate frail | 9 (9.5) | 46 (18.6) | 17 (23.9) | 19 (23.5) |
| Severely frail | 2 (2.1) | 1 (0.4) | 5 (7.0) | 5 (6.2) |
| Organ support during ICU stay – no. (%) |  |  |  |  |
| Renal replacement therapy | 35 (46.1) | 17 (7.9) | 40 (56.3) | 32 (34.8) |
| Extracorporeal membrane oxygenation | 10 (9.1) | 13 (4.3) | 15 (16.1) | 11 (10.2) |
| Non-invasive ventilation | 30 (30.0) | 52 (18.2) | 22 (26.2) | 17 (16.3) |
| Tracheostomy | 9 (11.8) | 12 (5.4) | 10 (14.3) | 5 (5.4) |
| Inotrope and/or vasopressor | 75 (97.4) | 174 (80.6) | 70 (98.6) | 83 (89.2) |
| Baseline WHODAS |  |  |  |  |
| Percentage | 13.5 (2.1 - 37.5) | 8.3 (0.0 - 35.4) | --- | --- |
| No disability | 39 (36.8) | 136 (45.3) | --- | --- |
| Mild disability | 28 (26.4) | 68 (22.7) | --- | --- |
| Moderate disability | 20 (18.9) | 51 (17.0) | --- | --- |
| Severe disability | 18 (17.0) | 44 (14.7) | --- | --- |
| Complete disability | 1 (0.9) | 1 (0.3) | --- | --- |
| Data are median (quartile 25% - quartile 75%) or No (%). Percentages may not total 100 because of rounding.  *APACHE: Acute Physiology and Chronic Health Evaluation; ICU: intensive care unit* | | | | |

| eTable 6 - Patient Characteristics According to Respondents and Non-Respondents at 6 Months | | | | | |
| --- | --- | --- | --- | --- | --- |
|  | **No Respondents**  **(*n* = 218)** | | **Respondents**  **(*n* = 448)** | |  |
|  | **Sepsis**  **(*n* = 64)** | **No Sepsis**  **(*n* = 154)** | **Sepsis**  **(*n* = 118)** | **No Sepsis**  **(*n* = 330)** |  |
| Age, years | 55.5 (42.7 - 69.9) | 54.5 (38.8 - 67.7) | 57.0 (42.9 - 68.2) | 58.5 (46.6 - 67.4) |  |
| Male gender – no. (%) | 35 (54.7) | 82 (53.2) | 76 (64.4) | 215 (65.2) |  |
| Body mass index, kg/m^2^ | 28.9 (24.0 - 30.8) | 26.3 (23.0 - 29.5) | 27.8 (23.9 - 33.0) | 26.8 (23.2 - 30.9) |  |
| Marital status – no. (%) |  |  |  |  |  |
| Separated or divorced | 1 (4.5) | 8 (16.7) | 6 (5.6) | 37 (12.2) |  |
| Living with a loved one | 13 (59.1) | 22 (45.8) | 62 (57.9) | 204 (67.1) |  |
| APACHE III | 71.0 (62.0 - 88.0) | 55.0 (41.0 - 73.0) | 71.0 (54.0 - 84.0) | 55.0 (43.0 - 70.0) |  |
| Type of admission – no. (%) |  |  |  |  |  |
| Medical | 49 (79.0) | 83 (54.6) | 89 (75.4) | 149 (46.0) |  |
| Surgical | 13 (21.0) | 69 (45.4) | 29 (24.6) | 175 (54.0) |  |
| Acute respiratory failure | 11 (20.0) | 3 (2.5) | 16 (14.0) | 12 (3.8) |  |
| Cardiac arrest | 2 (3.2) | 13 (8.7) | 7 (5.9) | 35 (10.8) |  |
| Diagnosis category – no. (%) |  |  |  |  |  |
| Cardiovascular | 12 (19.4) | 48 (31.2) | 26 (22.0) | 124 (37.6) |  |
| Gastrointestinal | 4 (6.5) | 20 (13.0) | 7 (5.9) | 25 (7.6) |  |
| Gynecological | 0 (0.0) | 1 (0.6) | 0 (0.0) | 1 (0.3) |  |
| Hematological | 0 (0.0) | 0 (0.0) | 0 (0.0) | 1 (0.3) |  |
| Metabolic | 0 (0.0) | 17 (11.0) | 3 (2.5) | 21 (6.4) |  |
| Musculoskeletal and skin | 2 (3.2) | 6 (3.8) | 2 (1.6) | 7 (2.1) |  |
| Neurological | 1 (1.6) | 10 (6.5) | 2 (1.7) | 17 (5.2) |  |
| Renal and genitourinary | 0 (0.0) | 1 (0.6) | 1 (0.8) | 4 (1.2) |  |
| Respiratory | 8 (12.9) | 25 (16.2) | 16 (13.6) | 73 (22.1) |  |
| Sepsis | 31 (50.0) | 0 (0.0) | 52 (44.1) | 0 (0.0) |  |
| Trauma | 4 (6.5) | 26 (16.9) | 9 (7.6) | 57 (17.3) |  |
| Co-existing disorders – no. (%) |  |  |  |  |  |
| Chronic respiratory failure |  |  |  |  |  |
| Lung transplant | 2 (3.3) | 7 (4.8) | 4 (3.4) | 33 (10.2) |  |
| Other | 2 (3.3) | 3 (2.1) | 3 (2.5) | 10 (3.1) |  |
| Chronic cardiovascular disease | 2 (3.3) | 8 (5.5) | 5 (4.2) | 20 (6.2) |  |
| Chronic liver disease | 2 (3.3) | 7 (4.8) | 4 (3.4) | 5 (1.5) |  |
| Chronic kidney disease | 0 (0.0) | 2 (1.4) | 2 (1.7) | 4 (1.2) |  |
| Chronic immune disease | 0 (0.0) | 2 (1.4) | 3 (2.5) | 1 (0.3) |  |
| Chronic immunosuppression | 5 (8.2) | 8 (5.5) | 7 (5.9) | 20 (6.2) |  |
| Diabetes | 19 (30.6) | 41 (28.5) | 48 (40.7) | 110 (33.3) |  |
| Hospital source of admission – no. (%) |  |  |  |  |  |
| Home | 38 (61.3) | 109 (72.2) | 67 (56.8) | 224 (69.3) |  |
| Other acute hospital not ICU | 12 (19.4) | 32 (21.2) | 26 (22.0) | 84 (26.0) |  |
| Other hospital ICU | 12 (19.4) | 7 (4.6) | 25 (21.2) | 14 (4.3) |  |
| Rehabilitation | 0 (0.0) | 2 (1. | 0 (0.0) | 1 (0.3) |  |
| Nursing home | 0 (0.0) | 0 (0.0) | 0 (0.0) | 0 (0.0) |  |
| Mental health | 0 (0.0) | 1 (0.7) | 0 (0.0) | 0 (0.0) |  |
| ICU source of admission – no. (%) |  |  |  |  |  |
| Emergency room | 21 (33.9) | 55 (36.2) | 33 (28.0) | 99 (30.7) |  |
| Operating room | 13 (21.0) | 66 (43.4) | 31 (26.3) | 173 (53.6) |  |
| Ward | 13 (21.0) | 15 (9.9) | 21 (17.8) | 22 (6.8) |  |
| Other hospital | 15 (24.2) | 15 (9.9) | 33 (27.9) | 28 (8.7) |  |
| Other ICU | 0 (0.0) | 1 (0.7) | 0 (0.0) | 1 (0.3) |  |
| Treatment limitation at ICU admission – no. (%) | 2 (3.5) | 1 (0.8) | 1 (0.9) | 0 (0.0) |  |
| Clinical frailty score at ICU admission – no. (%) |  |  |  |  |  |
| Non-frail | 42 (84.0) | 76 (79.2) | 84 (88.4) | 200 (81.0) |  |
| Mild-to-moderate frail | 7 (14.0) | 19 (19.8) | 9 (9.5) | 46 (18.6) |  |
| Severely frail | 1 (2.0) | 1 (1.0) | 2 (2.1) | 1 (0.4) |  |
| Organ support during ICU stay – no. (%) |  |  |  |  |  |
| Renal replacement therapy | 20 (46.5) | 9 (10.3) | 35 (46.1) | 17 (7.9) |  |
| Extracorporeal membrane oxygenation | 8 (15.4) | 3 (2.6) | 10 (9.1) | 13 (4.3) |  |
| Non-invasive ventilation | 17 (31.5) | 14 (13.2) | 30 (30.0) | 52 (18.2) |  |
| Tracheostomy | 3 (7.0) | 4 (4.5) | 9 (11.8) | 12 (5.4) |  |
| Inotrope and/or vasopressor | 42 (93.3) | 76 (86.4) | 75 (97.4) | 174 (80.6) |  |
| Baseline WHODAS |  |  |  |  |  |
| Percentage | 30.2 (10.4 - 59.4) | 29.2 (8.3 - 47.9) | 13.5 (2.1 - 37.5) | 8.3 (0.0 - 35.4) |  |
| No disability | 3 (15.0) | 11 (22.4) | 39 (36.8) | 136 (45.3) |  |
| Mild disability | 5 (25.0) | 12 (24.5) | 28 (26.4) | 68 (22.7) |  |
| Moderate disability | 6 (30.0) | 14 (28.6) | 20 (18.9) | 51 (17.0) |  |
| Severe disability | 6 (30.0) | 12 (24.5) | 18 (17.0) | 44 (14.7) |  |
| Complete disability | 0 (0.0) | 0 (0.0) | 1 (0.9) | 1 (0.3) |  |
| Data are median (quartile 25% - quartile 75%) or No (%). Percentages may not total 100 because of rounding.  *APACHE: Acute Physiology and Chronic Health Evaluation; ICU: intensive care unit* | | | | | |

| eTable 7 - Clinical Outcomes According to Presence of Sepsis During ICU Stay | | | | | | |
| --- | --- | --- | --- | --- | --- | --- |
|  |  |  | **Unadjusted Analysis** | | **Adjusted Analysis*** | |
|  | **Sepsis**  **(*n* = 282)** | **No Sepsis**  **(*n* = 606)** | **Absolute Difference**  **(95% CI)** | ***p* value** | **Absolute Difference**  **(95% CI)** | ***p* value** |
| Duration of ventilation, days | 5.5 (2.7 – 9.6) | 2.6 (1.5 – 4.9) | MD, 3.73 (2.81 to 4.64) | < 0.001 | MD, 3.07 (2.08 to 4.06) | < 0.001 |
| In survivors, days | 4.8 (2.5 – 8.3) | 2.4 (1.5 – 4.8) | MD, 2.91 (1.90 to 3.92) | < 0.001 | MD, 1.79 (0.72 to 2.89) | 0.001 |
| ICU length of stay, days | 9.8 (5.7 – 14.9) | 4.8 (3.0 – 8.7) | MD, 5.44 (4.14 to 6.74) | < 0.001 | MD, 4.73 (3.33 to 6.13) | < 0.001 |
| In survivors, days | 9.8 (5.7 – 14.8) | 4.9 (3.0 – 9.0) | MD, 4.96 (3.49 to 6.42) | < 0.001 | MD, 3.43 (1.88 to 5.03) | < 0.001 |
| Hospital length of stay, days | 20.5 (12.8 – 37.0) | 14.9 (8.4 – 25.7) | MD, 8.94 (5.92 to 11.96) | < 0.001 | MD, 9.55 (6.42 to 12.70) | < 0.001 |
| In survivors, days | 25.9 (16.0 – 43.2) | 16.1 (9.8 – 26.5) | MD, 12.63 (8.95 to 16.31) | < 0.001 | MD, 11.62 (7.81 to 15.51) | < 0.001 |
| Discharged home – no. (%) | 92 / 279 (33.0) | 315 / 592 (53.2) | RD, -20.23 (-26.94 to -13.31) | < 0.001 | RD, -12.20 (-19.17 to -5.24) | 0.001 |
| Mortality – no. (%) |  |  |  |  |  |  |
| ICU | 75 (26.6) | 75 (12.4) | RD, 14.22 (8.57 to 20.14) | < 0.001 | RD, 7.34 (1.96 to 12.71) | 0.008 |
| Hospital | 95 (33.7) | 102 (16.8) | RD, 16.86 (10.68 to 23.20) | < 0.001 | RD, 8.20 (2.22 to 13.98) | 0.007 |
| 90-day | 96 (34.0) | 117 (19.3) | RD, 14.74 (8.46 to 21.17) | < 0.001 | RD, 5.11 (-1.02 to 11.03) | 0.099 |
| 180-day | 100 (35.5) | 122 (20.1) | RD, 15.33 (8.97 to 21.82) | < 0.001 | RD, 5.64 (-0.49 to 11.66) | 0.072 |
| Data are median (quartile 25% - quartile 75%) or No (%). Percentages may not total 100 because of rounding. Denominators are shown when the overall sample size was not available.  *APACHE: Acute Physiology and Chronic Health Evaluation; ICU: intensive care unit; CI: confidence interval; MD: mean difference; RD: risk difference.*  * All analyses adjusted for age, sex, ICU admission source, APACHE III score, type of admission (medical vs. surgical), lung transplant patients, trauma, creatinine, heart rate, mean arterial pressure, presence of chronic cardiovascular disease and with centers included as random effect. | | | | | | |

| eTable 8 - Baseline Function and Health Status According to the Presence of Sepsis* | | | | |
| --- | --- | --- | --- | --- |
|  | **Sepsis**  **(*n* = 282)** | **No Sepsis**  **(*n* = 606)** | **Absolute Difference**  **(95% CI)** | ***p* value** |
| WHODAS score | 25.7 ± 27.4 | 20.7 ± 24.1 | MD, 5.00 (-0.09 to 10.09) | 0.054 |
| Disability | 52 / 127 (40.9) | 122 / 350 (34.9) | RD, 6.09 (-3.67 to 16.06) | 0.228 |
| EuroQol-visual analogue scale | 58.2 ± 31.2 | 61.0 ± 29.7 | MD, -2.80 (-8.89 to 3.29) | 0.367 |
| EQ-5D-5L™ utility | 0.6 ± 0.4 | 0.6 ± 0.4 | MD, -0.03 (-0.10 to 0.04) | 0.402 |
| No problem with anxiety | 76 / 130 (58.5) | 197 / 353 (55.8) | RD, 2.65 (-7.35 to 12.44) | 0.600 |
| No problem with mobility | 61 / 130 (46.9) | 207 / 353 (58.6) | RD, -11.72 (-21.61 to -1.70) | 0.022 |
| No problem with pain | 62 / 130 (47.7) | 188 / 353 (53.3) | RD, -5.57 (-15.53 to 4.47) | 0.277 |
| No problem with personal care | 81 / 130 (62.3) | 227 / 353 (64.3) | RD, -2.00 (-11.83 to 7.53) | 0.687 |
| No problem with usual activities | 59 / 130 (45.4) | 183 / 353 (51.8) | RD, -6.46 (-16.37 to 3.59) | 0.207 |
| Data are mean ± standard deviation or No (%). Percentages may not total 100 because of rounding. Denominators are shown when the overall sample size was not available.  *MD is mean difference; RD: risk difference.*  * 675 patients were alive at 3 months, the moment of assessment (186 in the sepsis and 489 in the no sepsis group) | | | | |

|  | | | | | | | | | | |
| --- | --- | --- | --- | --- | --- | --- | --- | --- | --- | --- |
| eTable 9 - Long-Term Outcomes According to the Presence of Sepsis (Unadjusted Analyses) | | | | | | | | | |  |
|  | **At 3 Months*** | | | | **At 6 Months**** | | | |  |  |
|  | **Sepsis**  **(*n* = 282)** | **No Sepsis**  **(*n* = 606)** | **Absolute Difference^a^**  **(95% CI)** | ***p* value** | **Sepsis**  **(*n* = 282)** | **No Sepsis**  **(*n* = 606)** | **Absolute Difference^a^**  **(95% CI)** | ***p* value** | ***p* for Interaction^b^** |  |
| WHODAS score, % | 31.8 ± 23.5 | 24.0 ± 22.0 | MD, 7.89 (3.51 to 12.28) | < 0.001 | 26.1 ± 22.1 | 21.5 ± 21.1 | MD, 3.68 (-0.8 to 8.16) | 0.107 | 0.027 |  |
| Disability - no. (%) | 73 / 129 (56.6) | 145 / 348 (41.7) | RD, 13.89 (4.03 to 23.74) | 0.006 | 52 / 118 (44.1) | 126 / 330 (38.2) | RD, 4.33 (-5.83 to 14.48) | 0.403 | 0.067 |  |
| New disability^¥^ - no. (%) | 50 / 126 (39.7) | 114 / 344 (33.1) | RD, 6.39 (-3.38 to 16.16) | 0.199 | 42 / 106 (39.6) | 106 / 300 (35.3) | RD, 1.45 (-8.87 to 11.77) | 0.783 | 0.307 |  |
| New disability or death - no. (%) | 146 / 222 (65.8) | 231 / 461 (50.1) | RD, 15.66 (7.82 to 23.51) | < 0.001 | 142 / 206 (68.9) | 228 / 422 (54.0) | RD, 12.92 (4.93 to 20.92) | 0.002 | 0.362 |  |
| EuroQol-visual analogue scale | 61.0 ± 24.3 | 67.4 ± 22.9 | MD, -6.49 (-10.89 to -2.09) | 0.004 | 66.1 ± 20.7 | 71.4 ± 20.1 | MD, -4.4 (-8.95 to 0.15) | 0.058 | 0.389 |  |
| EQ-5D-5L™ utility | 0.6 ± 0.3 | 0.7 ± 0.3 | MD, -0.09 (-0.15 to -0.03) | 0.002 | 0.7 ± 0.3 | 0.7 ± 0.3 | MD, -0.06 (-0.12 to 0) | 0.036 | 0.285 |  |
| No problem with anxiety | 63 / 129 (48.8) | 195 / 349 (55.9) | RD, -6.96 (-16.88 to 2.97) | 0.169 | 71 / 118 (60.2) | 196 / 332 (59.0) | RD, 1.64 (-8.59 to 11.87) | 0.753 | 0.109 |  |
| No problem with mobility | 49 / 129 (38.0) | 204 / 349 (58.5) | RD, -20.22 (-30 to -10.45) | < 0.001 | 63 / 118 (53.4) | 220 / 332 (66.3) | RD, -12.5 (-22.6 to -2.39) | 0.015 | 0.175 |  |
| No problem with pain | 51 / 129 (39.5) | 165 / 349 (47.3) | RD, -8.32 (-18.34 to 1.7) | 0.104 | 56 / 118 (47.5) | 171 / 332 (51.5) | RD, -2.6 (-12.96 to 7.76) | 0.623 | 0.326 |  |
| No problem with personal care | 66 / 129 (51.2) | 217 / 349 (62.2) | RD, -11.00 (-20.67 to -1.33) | 0.026 | 64 / 118 (54.2) | 230 / 332 (69.3) | RD, -14.07 (-24.05 to -4.09) | 0.006 | 0.568 |  |
| No problem with usual activities | 34 / 129 (26.4) | 117 / 349 (33.5) | RD, -8.06 (-17.62 to 1.5) | 0.098 | 37 / 118 (31.4) | 139 / 332 (41.9) | RD, -8.58 (-18.48 to 1.31) | 0.089 | 0.927 |  |
| Unemployed due to health | 64 / 129 (49.6) | 162 / 348 (46.6) | RD, 2.78 (-7.1 to 12.65) | 0.581 | 52 / 118 (44.1) | 135 / 334 (40.4) | RD, 2.59 (-7.52 to 12.69) | 0.616 | 0.966 |  |
| IES-R | --- | --- | --- | --- | 12.3 ± 14.4 | 8.5 ± 12.5 | MD, 3.83 (0.08 to 7.59) | 0.045 | --- |  |
| Post-traumatic stress disorder | --- | --- | --- | --- | 8 / 62 (12.9) | 9 / 184 (4.9) | RD, 8.01 (0.72 to 15.31) | 0.032 | --- |  |
| IADL | --- | --- | --- | --- | 6.5 ± 2.1 | 7.1 ± 1.7 | MD, -0.62 (-1.00 to -0.24) | 0.001 | --- |  |
| Fully independent | --- | --- | --- | --- | 61 / 117 (52.1) | 214 / 327 (65.4) | RD, -13.31 (-23.53 to -3.08) | 0.011 | --- |  |
| MoCA-BLIND | --- | --- | --- | --- | 18.6 ± 2.8 | 18.5 ± 3.1 | MD, 0.08 (-0.80 to 0.96) | 0.860 | --- |  |
| Cognitive dysfunction | --- | --- | --- | --- | 18 / 61 (29.5) | 50 / 174 (28.7) | RD, 0.77 (-12.58 to 14.12) | 0.909 | --- |  |
| HADS anxiety | --- | --- | --- | --- | 4.7 ± 4.7 | 4.3 ± 4.3 | MD, 0.46 (-0.72 to 1.65) | 0.444 | --- |  |
| Anxiety | --- | --- | --- | --- | 19 / 73 (26.0) | 47 / 212 (22.2) | RD, 3.86 (-7.44 to 15.16) | 0.502 | --- |  |
| HADS depression | --- | --- | --- | --- | 4.1 ± 3.6 | 3.6 ± 3.8 | MD, 0.58 (-0.41 to 1.58) | 0.249 | --- |  |
| Depression | --- | --- | --- | --- | 15 / 73 (20.5) | 36 / 208 (17.3) | RD, 3.24 (-7.11 to 13.59) | 0.538 | --- |  |
| Financial distress | 2.7 ± 3.4 | 2.4 ± 3.3 | MD, 0.38 (-0.3 to 1.06) | 0.270 | 2.2 ± 3.3 | 1.8 ± 3.0 | MD, 0.37 (-0.35 to 1.1) | 0.314 | 0.984 |  |
| Data are mean ± standard deviation or No (%). Percentages may not total 100 because of rounding. Denominators are shown when the overall sample size was not available.  *MD is mean difference; RD is risk difference.*  * 675 patients were alive at 3 months (186 in the sepsis and 489 in the no sepsis group).  ** 666 patients were alive at 3 months (182 in the sepsis and 484 in the no sepsis group).  ^a^ all models are mixed-effect models considering the moment of measurement, group, as well as the group x time interaction as fixed effect. Moment of measurement was treated as a categorical variable and random intercept for patients was included to account for the dependency of repeated measures. Between-group comparisons at each timepoint was estimate with the appropriate contrasts from the model and using a Holm-Bonferroni method to adjust for multiplicity. In all models the no sepsis group was used as reference (OR > 1 represents increased risk is septic patients and MD > 1 represents increase in the score in septic patients).  ^b^ *p* value for interaction between sepsis group and moment of measurement.  ^¥^ new disability defined as a change of WHODAS ≥ 10%. | | | | | | | | | |  |

| eTable 10 - Long-Term Outcomes According to the Presence of Sepsis After Multiple Imputation | | | | | | | | | | |
| --- | --- | --- | --- | --- | --- | --- | --- | --- | --- | --- |
|  | **Unadjusted Analyses** | | | | | **Adjusted Analyses*** | | | | |
|  | **At 3 Months** | | **At 6 Months** | |  | **At 3 Months** | | **At 6 Months** | |  |
|  | **Effect Estimate^a^**  **(95% CI)** | ***p* value** | **Effect Estimate^a^**  **(95% CI)** | ***p* value** | ***p* for Interaction^b^** | **Effect Estimate^a^**  **(95% CI)** | ***p* value** | **Effect Estimate^a^**  **(95% CI)** | ***p* value** | ***p* for Interaction^b^** |
| WHODAS score, % | MD, 6.43 (-0.23 to 13.10) | 0.058 | MD, 4.82 (-3.34 to 13.00) | 0.246 | 0.566 | MD, 2.75 (-5.27 to 10.77) | 0.501 | MD, 1.26 (-5.92 to 8.44) | 0.730 | 0.614 |
| Disability | RD, 10.87 (-1.81 to 23.60) | 0.093 | RD, 4.27 (-5.28 to 13.80) | 0.381 | 0.314 | RD, 6.68 (-6.53 to 19.90) | 0.321 | RD, 0.25 (-10.02 to 10.50) | 0.962 | 0.342 |
| New disability^¥^ | RD, 2.69 (-10.70 to 16.00) | 0.693 | RD, -0.30 (-16.20 to 15.60) | 0.970 | 0.656 | RD, 0.82 (-10.40 to 12.00) | 0.885 | RD, -2.70 (-16.30 to 10.90) | 0.697 | 0.578 |
| EuroQol-visual analogue scale | MD, -4.37 (-9.33 to 0.59) | 0.085 | MD, -4.15 (-9.88 to 1.570 | 0.154 | 0.928 | MD, -2.43 (-7.06 to 2.21) | 0.305 | MD, -2.15 (-7.33 to 3.03) | 0.416 | 0.907 |
| EQ-5D-5L™ utility | MD, -0.04 (-0.20 to 0.13) | 0.656 | MD, -0.06 (-0.22 to 0.09) | 0.456 | 0.668 | MD, -0.04 (-0.14 to 0.06) | 0.402 | MD, -0.05 (-0.15 to 0.04) | 0.260 | 0.804 |
| No problem with anxiety | RD, -4.15 (-17.60 to -9.29) | 0.544 | RD, 1.09 (-8.78 to 10.95) | 0.829 | 0.396 | RD, -6.09 (-20.80 to 8.65) | 0.418 | RD, -0.98 (-11.80 to 9.80) | 0.857 | 0.396 |
| No problem with mobility | RD, -15.50 (-25.70 to -5.29) | 0.003 | RD, -11.30 (-21.30 to -1.32) | 0.026 | 0.514 | RD, -11.97 (-21.60 to -2.36) | 0.014 | RD, -7.44 (-20.00 to 5.08) | 0.244 | 0.487 |
| No problem with pain | RD, -2.42 (-16.30 to 11.40) | 0.732 | RD, -2.96 (-16.10 to 10.10) | 0.658 | 0.928 | RD, -0.98 (-14.80 to 12.80) | 0.888 | RD, -1.76 (-16.30 to 12.80) | 0.812 | 0.898 |
| No problem with personal care | RD, -6.01 (-16.90 to 4.91) | 0.280 | RD, -10.78 (-21.40 to -0.01) | 0.047 | 0.405 | RD, -1.78 (-12.40 to 8.83) | 0.742 | RD, -6.40 (-15.50 to 2.68) | 0.167 | 0.431 |
| No problem with usual activities | RD, -5.96 (-18.40 to 6.45) | 0.346 | RD, -6.94 (-19.90 to 6.07) | 0.296 | 0.864 | RD, -2.52 (-15.40 to 10.40) | 0.702 | RD, -3.39 (-18.50 to 11.70) | 0.659 | 0.879 |
| Unemployed due to health | RD, 2.37 (-6.85 to 11.60) | 0.614 | RD, 3.05 (-6.94 to 13.00) | 0.549 | 0.884 | RD, -0.77 (-11.00 to 9.48) | 0.881 | RD, -0.06 (-11.50 to 11.41) | 0.991 | 0.876 |
| IES-R | --- | --- | MD, 1.59 (-8.43 to 11.62) | 0.713 | --- | --- | --- | MD, 0.54 (-9.39 to 10.49) | 0.899 | --- |
| Post-traumatic stress disorder | --- | --- | RD, 5.98 (-14.21 to 26.17) | 0.505 | --- | --- | --- | RD, 3.74 (-16.04 to 23.53) | 0.674 | --- |
| IADL | --- | --- | MD, -0.38 (-0.93 to 0.16) | 0.152 | --- | --- | --- | MD, -0.46 (-1.08 to 0.16) | 0.135 | --- |
| Fully independent | --- | --- | RD, -9.26 (-20.24 to 1.71) | 0.095 | --- | --- | --- | RD, -9.57 (-24.78 to 5.64) | 0.196 | --- |
| MoCA-BLIND | --- | --- | MD, -0.41 (-1.76 to 0.94) | 0.507 | --- | --- | --- | MD, -0.16 (-1.40 to 1.07) | 0.781 | --- |
| Cognitive dysfunction | --- | --- | RD, 7.95 (-14.53 to 30.45) | 0.422 | --- | --- | --- | RD, 3.04 (-22.49 to 28.57) | 0.780 | --- |
| HADS anxiety | --- | --- | MD, 0.32 (-1.80 to 2.45) | 0.724 | --- | --- | --- | MD, 0.66 (-1.35 to 2.66) | 0.466 | --- |
| Anxiety | --- | --- | RD, 0.65 (-25.82 to 27.13) | 0.952 | --- | --- | --- | RD, 0.87 (-22.84 to 24.60) | 0.931 | --- |
| HADS depression | --- | --- | MD, 0.06 (-1.04 to 1.15) | 0.911 | --- | --- | --- | MD, -0.05 (-0.88 to 0.79) | 0.904 | --- |
| Depression | --- | --- | RD, 0.51 (-18.57 to 19.59) | 0.951 | --- | --- | --- | RD, 1.67 (-14.73 to 18.07) | 0.827 | --- |
| Financial distress | MD, 0.07 (-0.67 to 0.82) | 0.845 | MD, 0.22 (-0.47 to 0.93) | 0.522 | 0.746 | MD, -0.07 (-0.77 to 0.63) | 0.633 | MD, 0.08 (-0.66 to 0.82) | 0.832 | 0.753 |
| *MD is mean difference; OR is odds ratio.*  ^a^ effect estimate is mean difference for continuous variable and odds ratio for binary variables. All models are mixed-effect models considering the moment of measurement, group, as well as the group x time interaction as fixed effect. Moment of measurement was treated as a categorical variable and random intercepts for patients and centers were included to account for the dependency of repeated measures and clustering of the data. Between-group comparisons at each timepoint was estimate with the appropriate contrasts from the model and using a Holm-Bonferroni method to adjust for multiplicity. In all models the no sepsis group was used as reference (OR > 1 represents increased risk is septic patients and MD > 1 represents increase in the score in septic patients).  * All models were adjusted by age, APACHE III score, type of admission (medical vs. surgical), lung transplant patients, presence of chronic cardiovascular disease, and ICU length of stay. Whenever available, models were further adjusted by the baseline value of the outcome of interest as fixed effect.  ^b^ *p* value for interaction between sepsis group and moment of measurement  ^¥^ new disability defined as a change of WHODAS ≥ 10%. | | | | | | | | | | |

# eFigure 1 - Flowchart of Inclusion

**
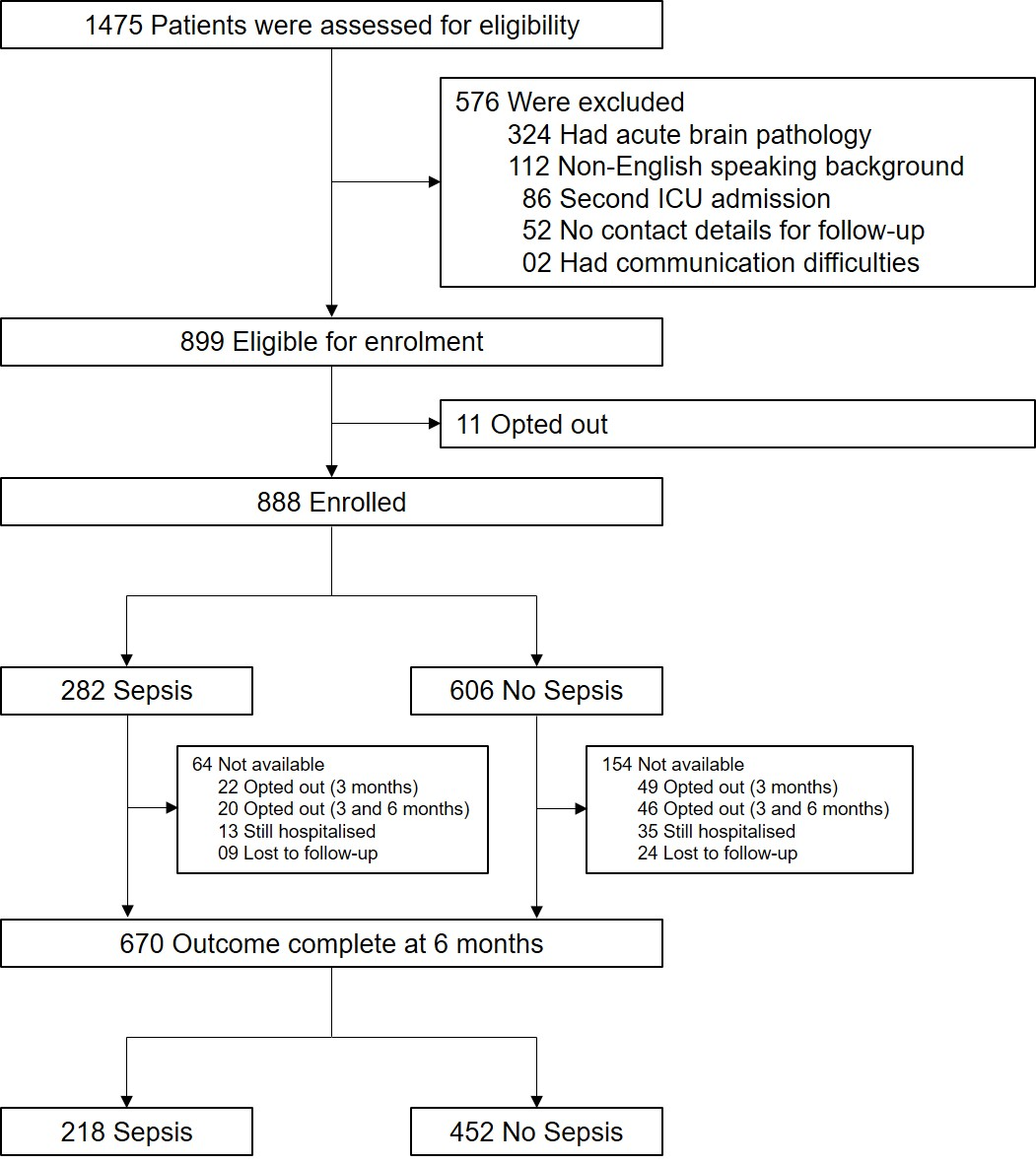
**

# eFigure 2 - Trajectory of Long-Term Outcomes in Patients with and Without Sepsis

**
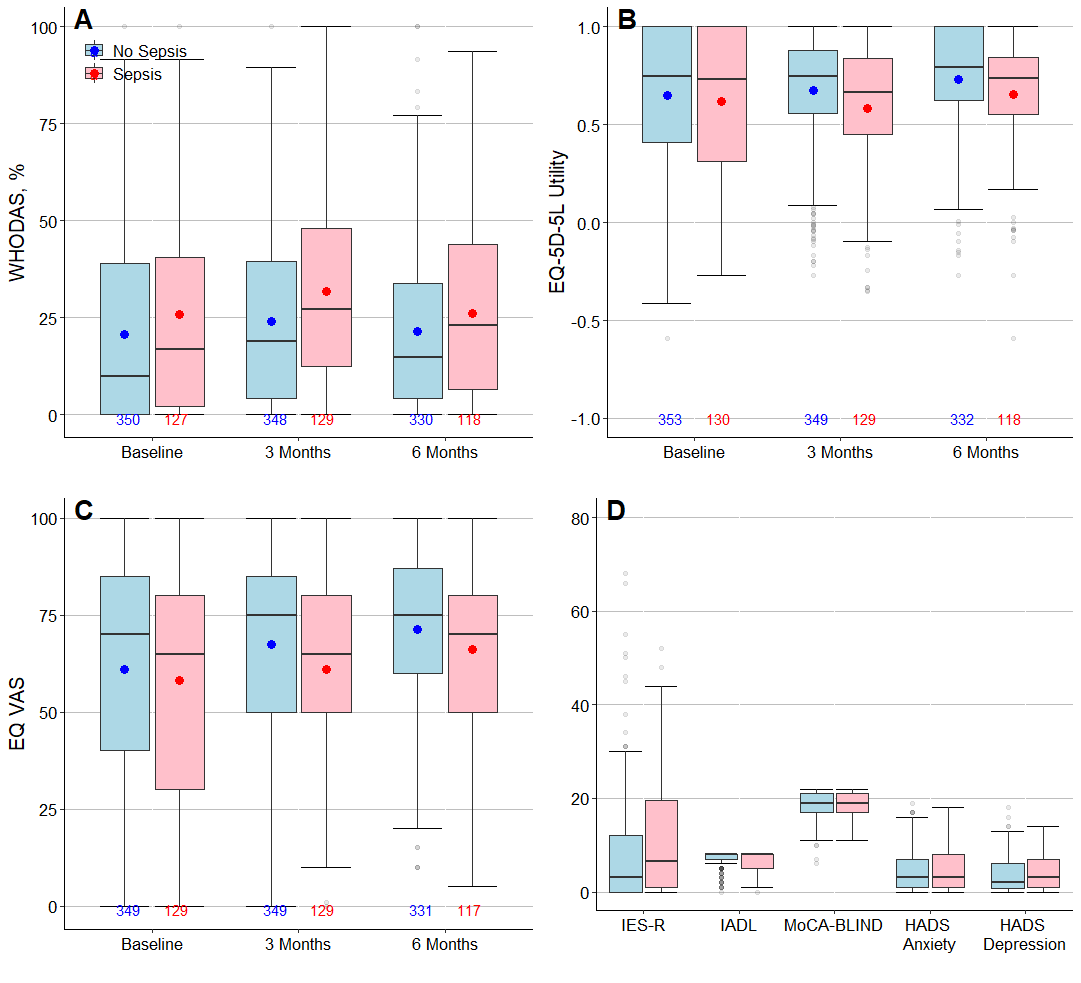
**

Panel A, B, C and D: Boxes represent median and interquartile range. Whiskers extend 1.5 times the interquartile range beyond the first and third quartiles per the conventional Tukey method. Transparent circles beyond the whiskers represent outliers. Filled circles are the mean.

Abbreviations: WHODAS, WHO Disability Assessment Schedule 2.0; IES-R, Impact of Event Scale–Revised; IADL, Instrumental Activities of Daily Living; MoCA-BLIND, Montreal Cognitive Assessment.

# eFigure 3 - Incidence of Disability, Problems Related to the EQ-5D-5L, Unemployment due to Health Problems at Baseline and 3 Months (an increase in disability and unemployed indicates a worse outcome, an increase in “no problem” indicates a better outcome)

**
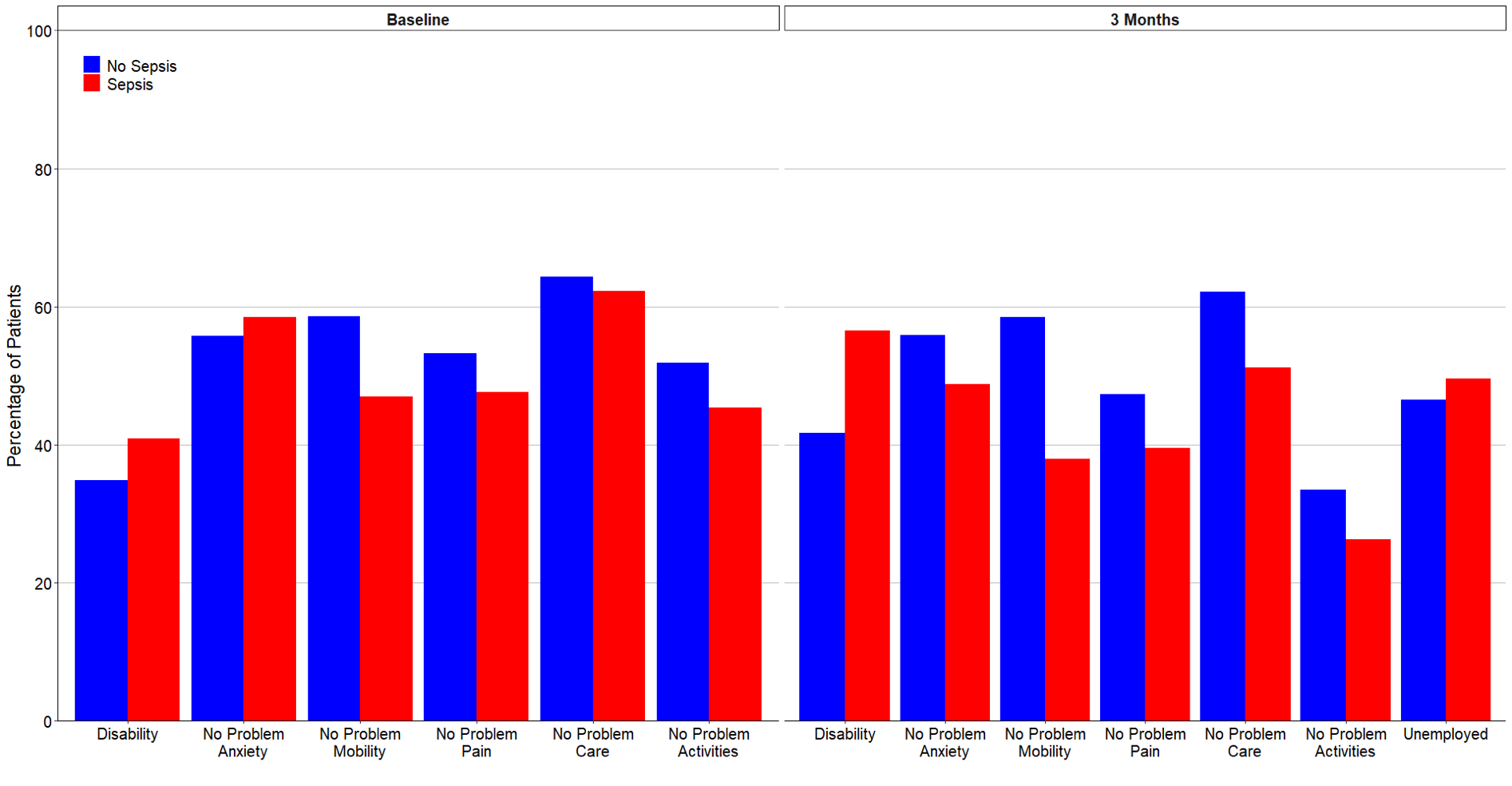
**

Disability defined as WHODAS ≥ 25%, components of EQ-5D-5L™ (according to no development of problems), did not return to work due to health problems (based on WHODAS II work).

# eFigure 4 - Incidence of Disability, Problems Related to the EQ-5D-5L, Unemployment due to Health Problems, Post-Traumatic Stress Disorder, Independence and Cognitive Dysfunction at 6 Months(an increase in disability, cognitive dysfunction and unemployed indicates a worse outcome, an increase in “no problem” indicates a better outcome)

**
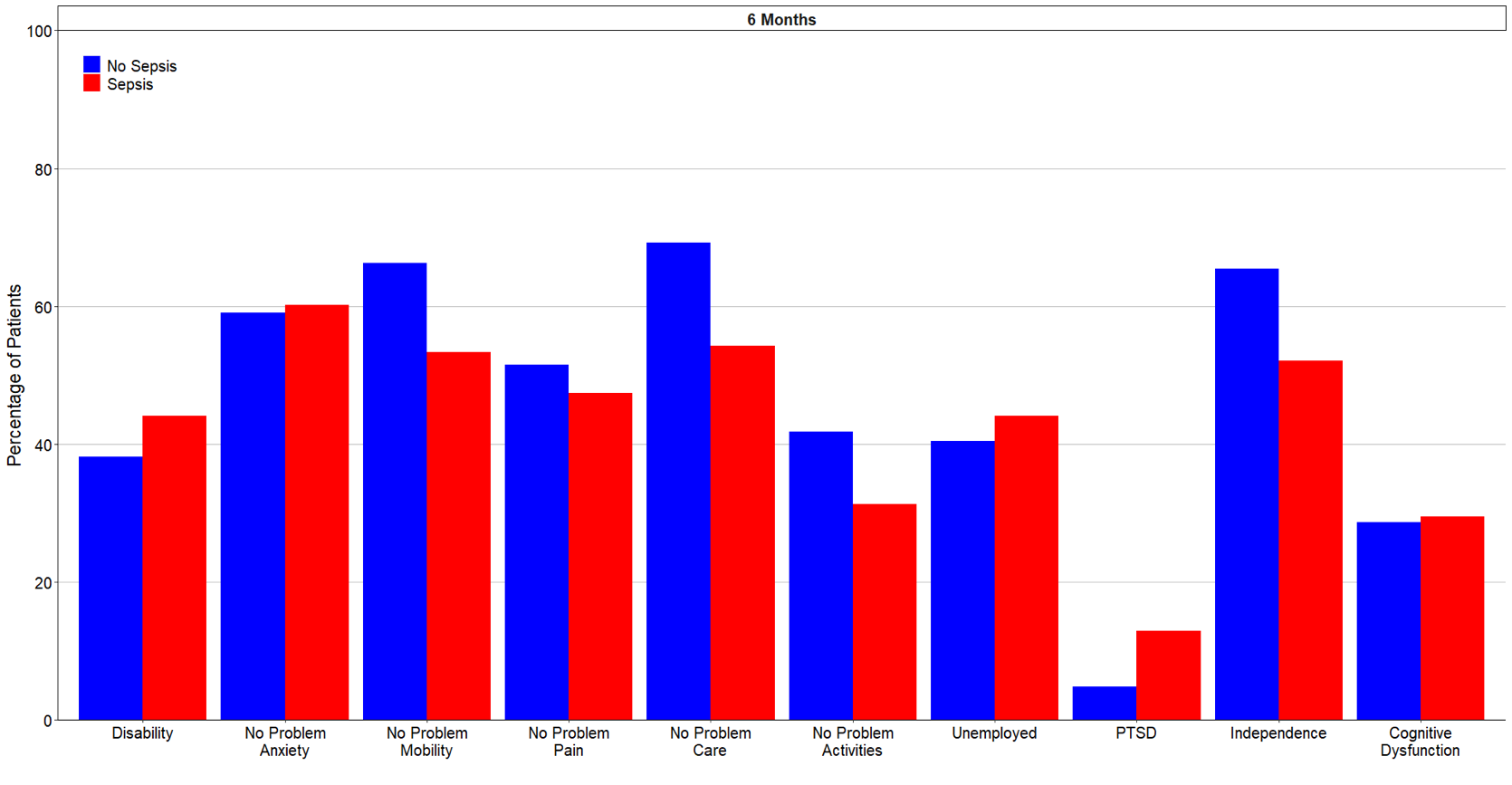
**

Disability defined as WHODAS ≥ 25%, components of EQ-5D-5L™ (according to no development of problems), significant anxiety (defined as HADS anxiety ≥ 8), significant depression (defined as HADS depression ≥ 8), did not return to work due to health problems (based on WHODAS II work), post-traumatic stress disorder (defined as IES-R ≥ 33), fully independent (defined as IADL = 8),

and cognitive dysfunction (defined as MoCA-BLIND < 18).
